# The (cost‐)effectiveness of preventive, integrated care for community‐dwelling frail older people: A systematic review

**DOI:** 10.1111/hsc.12571

**Published:** 2018-04-17

**Authors:** Wilhelmina Mijntje Looman, Robbert Huijsman, Isabelle Natalina Fabbricotti

**Affiliations:** ^1^ Department Health Services Management & Organisation Erasmus School of Health Policy & Management Erasmus University Rotterdam The Netherlands

**Keywords:** community‐dwelling frail older people, cost‐effectiveness, effectiveness, integrated care, prevention

## Abstract

Integrated care is increasingly promoted as an effective and cost‐effective way to organise care for community‐dwelling frail older people with complex problems but the question remains whether high expectations are justified. Our study aims to systematically review the empirical evidence for the effectiveness and cost‐effectiveness of preventive, integrated care for community‐dwelling frail older people and close attention is paid to the elements and levels of integration of the interventions. We searched nine databases for eligible studies until May 2016 with a comparison group and reporting at least one outcome regarding effectiveness or cost‐effectiveness. We identified 2,998 unique records and, after exclusions, selected 46 studies on 29 interventions. We assessed the quality of the included studies with the Effective Practice and Organization of Care risk‐of‐bias tool. The interventions were described following Rainbow Model of Integrated Care framework by Valentijn. Our systematic review reveals that the majority of the reported outcomes in the studies on preventive, integrated care show no effects. In terms of health outcomes, effectiveness is demonstrated most often for seldom‐reported outcomes such as well‐being. Outcomes regarding informal caregivers and professionals are rarely considered and negligible. Most promising are the care process outcomes that did improve for preventive, integrated care interventions as compared to usual care. Healthcare utilisation was the most reported outcome but we found mixed results. Evidence for cost‐effectiveness is limited. High expectations should be tempered given this limited and fragmented evidence for the effectiveness and cost‐effectiveness of preventive, integrated care for frail older people. Future research should focus on unravelling the heterogeneity of frailty and on exploring what outcomes among frail older people may realistically be expected.

**What is known about the topic**

- Integrated care is perceived as a promising solution for frail older people with complex problems to “age in place”.
- Despite the high expectations, a (recent) systematic review on the (cost)‐effectiveness of preventive, integrated care interventions for community‐dwelling frail older people is lacking.

**What this paper adds**

- The evidence for the (cost‐) effectiveness of preventive, integrated care is limited since the majority of reported outcomes show no effect and evidence is fragmented because populations, interventions and evaluation studies differ substantially.
- No clear relationship exists between (cost‐)effectiveness and specific preventive, integrated care elements or levels of integration.
- Researchers in integrated care should be more aware of the underlying principles of integrated care: they should integrate their research, consider continuity and differentiate between frail older people.

## 1 INTRODUCTION

Integrated care is increasingly promoted as an effective way to organise care for community‐dwelling frail older people. Societal developments such as population ageing and rising care costs have led to more frail older people with complex problems to “age in place” (Wiles, Leibing, Guberman, Reeve, & Allen, 2012). Their complex problems in the physical, psychological or social domain cannot be adequately addressed by a single primary care professional and require co‐ordination and multidisciplinary collaboration. A solution is found in integrated care which is defined as an organisational process of co‐ordination that seeks to achieve seamless and continuous care, tailored to the patient's needs and based on a holistic view of the patient (Mur‐Veeman, Hardy, Steenbergen, & Wistow, 2003). Integrated care is proclaimed to pursue a wide range of aims such as improving the quality of care and consumer satisfaction, enhancing clinical results, quality of life, system efficiency and cost‐effectiveness (Kodner & Spreeuwenberg, 2002). Professionals, policy makers and researchers consider integrated care as a complex phenomenon and promising solution. In literature, conceptual frameworks have been developed to enhance the understanding of integrated care (Valentijn, Schepman, Opheij, & Bruijnzeels, 2013). Several integrated care interventions for frail older people have been developed (Oliver, Foot, & Humphries, 2014) and much effort has been put into evaluating the effectiveness of these interventions (Evers & Paulus, 2015).

Despite the widespread interest in integrated care, a systematic review of integrated care interventions for community‐dwelling frail older people is lacking. Previous reviews have concentrated on specific interventions such as home‐visiting programmes (Elkan et al., 2001; Stuck, Egger, Hammer, Minder, & Beck, 2002) and case management (Stokes et al., 2015; You, Dunt, Doyle, & Hsueh, 2012) or have focused on other target groups such as older patients with chronic diseases (Ouwens, Wollersheim, Hermens, Hulscher, & Grol, 2005) and older people in general (Johri, Beland, & Bergman, 2003). Our aim is to systematically review the empirical evidence on the effectiveness and cost‐effectiveness of preventive, integrated care for frail older people in the community. Hence, our study makes five main contributions.

First, we focus explicitly on integrated care for community‐dwelling frail older people. Frailty is a specific condition that differs from chronic diseases (Fried et al., 2001) and chronological age (Slaets, 2006). Frailty refers to a dynamic state affecting an individual who experiences loss in one or more domains of human functioning (physical, psychological, social). This loss is influenced by a range of variables that increase the risk of adverse outcomes (Gobbens, Luijkx, Wijnen‐Sponselee, & Schols, 2010; Lacas & Rockwood, 2012). Other reviews focused on frail older people but their eligibility criteria were based on chronological age (Eklund & Wilhelmson, 2009; Johri et al., 2003). Focusing on community‐dwelling frail older people implies that the integrated care interventions are based in primary care which provides integrated, accessible healthcare services by clinicians who are accountable for addressing a large majority of personal healthcare needs, developing a sustained partnership with patients, and practicing in the context of family and community (Vanselow, Donaldson, & Yordy, 1995).

Second, our review provides insight into the value of prevention in integrated care interventions for frail older people, whereas previous systematic reviews have not paid explicit attention to the preventive component in integrated care (Eklund & Wilhelmson, 2009). Frailty should be prevented in order to reduce the risk of adverse outcomes such as health problems and disability (Fried et al., 2001), poor quality of life (Gobbens & van Assen, 2014) and crisis situations (Vedel et al., 2009). Prevention of frailty is also important to avoid or delay institutionalisation, thereby fulfilling an essential aim of national health policies. Therefore, it is important to incorporate prevention into integrated care interventions, including screening for frailty and comprehensive geriatric assessments (Oliver et al., 2014).

Third, our systematic review includes all quantitative designs with a control group and is not limited to randomised controlled trials. Although randomised controlled trials are known to provide strong evidence, their use is questioned for complex interventions (Clark, 2001). Integrated care interventions in primary care particularly illustrate the difficulties with randomised controlled trials because randomisation of participants to a general practitioner (GP) is almost impossible.

Fourth, our review incorporates economic evaluations of integrated care interventions for frail older people. Cost‐effectiveness is an important aim of integrated care (Kodner & Spreeuwenberg, 2002) and economic evaluations of integrated care for frail older people have recently generated considerable research interest (Evers & Paulus, 2015). Due to budget constraints and population ageing, health and social care expenditures are under pressure. Therefore, it is relevant to explore whether integrated care with a preventive component can put the available resources to optimal use.

Finally, we relate the effectiveness and cost‐effectiveness with the specific content of the preventive, integrated care interventions. In the current fragmented healthcare systems, achieving seamless and continuous care tailored to the needs of frail older people is complex. Integration could be pursued at different levels and with different strategies such as comprehensive geriatric assessments, multidisciplinary teams or organisational integration (Kodner & Spreeuwenberg, 2002; Valentijn et al., 2013). The assumption is that a higher level of integration leads to better outcomes (Kodner & Spreeuwenberg, 2002); however, it still remains unclear what specific bundles of integrated care lead to specific outcomes (Eklund & Wilhelmson, 2009; Kodner, 2009). Therefore, the preventive integrated care interventions will be analysed following the taxonomy of the Rainbow Model of Integrated Care; a conceptual framework for integrated care from a primary care perspective (Valentijn et al., 2013).

## 2 METHODS

The methods and results of this systematic review are reported according to PRISMA guidelines (Moher, Liberati, Tetzlaff, & Altman, 2009).

### 2.1 Search strategy

We searched nine databases, including Embase, Medline (Ovid), Web‐of‐Science, CINAHL (EBSCO), PsycINFO (Ovid), Cochrane, PubMed publisher, ProQuest (ABI Inform, Dissertations) and Google Scholar. The search terms were discussed with a medical librarian who is a specialist in conducting and designing searches for systematic reviews (Bramer, Giustini, Kramer, & Anderson, 2013). The main search terms were “integrated health care system,” “frail older people” and “primary care.” The complete Embase search strategy is presented in Appendix S1. Besides Boolean operators AND and OR, we used the proximity operators NEAR and NEXT so that terms within a certain reach were also detected in the search. The search was done in August 2015 and updated in May 2016.

### 2.2 Eligibility criteria

Box 1 presents the eligibility criteria of our systematic review.

Box 1 **Eligibility criteria**

1

**Table:** 

Inclusion criteria Population: community‐dwelling frail older people. Excluded: selecting participants on age, having a chronic condition, or hospitalised or institutionalised older people. Intervention: integrated care intervention with preventive component based in primary care. Comparison group: community‐dwelling frail older people receiving care as usual. Outcome: >1 outcome regarding the effectiveness for frail older people or the cost‐effectiveness of the intervention. Study designs: quantitative empirical studies with a control group
Exclusion criteria: policy intervention (at regional or national level) non‐English studies non‐peer reviewed studies

### 2.3 Study selection

After removing duplicates, one reviewer screened the titles of all articles. Then two reviewers independently screened the remaining abstracts according to the inclusion and exclusion criteria. Any disagreements over abstracts were discussed until the reviewers reached a consensus. The remaining full texts were assessed for eligibility by one reviewer. All full texts that met the inclusion criteria or where doubts arose were discussed with the second reviewer. A reference check was performed on all included full texts.

### 2.4 Data extraction

All included full texts were summarised, focusing on the study methods, the intervention and its outcomes. The methods of each study were described according to inclusion criteria (definition of frailty), study design, types of outcomes, sample size and country. The interventions are presented following the taxonomy of the Rainbow Model of Integrated Care (Valentijn et al., 2013). The elements of each intervention are distinguished according to the micro, meso and macro levels of integration described by Valentijn. The micro level consists of service integration in which the following elements are distinguished: assessment; care plan; follow‐up; and single entry point. The meso level includes professional integration (with four elements: the focal organisation of the intervention; the role of the GP, team composition and education professionals) and organisational integration. The macro level consists of financial integration. These three levels are connected by normative integration and functional integration (with two elements: co‐ordination and information system). Additional information is provided about the role of the informal caregiver and prevention in the interventions.

Five outcome categories are presented in subsequent tables: health outcomes, outcomes regarding informal caregivers and professionals, process outcomes, healthcare utilisation and cost‐effectiveness. The results for the outcomes are presented as follows: (+: significant outcome in favour of the intervention, 0: no significant outcome; −: significant outcome in favour of the control group; +/− significant outcome both in favour of the intervention and the control group within one category; NS: outcome not tested for significance). Outcomes are presented at the level of the intervention, so the results of studies reporting on the same intervention are combined. The number of statistically significant results has been counted.

### 2.5 Quality assessment

The quality of the included studies was assessed with the Effective Practice and Organization of Care (EPOC) risk‐of‐bias tool for studies with a separate control group (EPOC, 2015). This quality assessment tool is the most suitable to assess the included studies because our systematic review was not restricted to randomised controlled trials. The EPOC comprises nine standard criteria, including generation and concealment of allocation, similarity of outcome and baseline measures, adequacy of addressing missing outcome date, prevention of knowledge of allocated intervention, protection against contamination, selective outcome reporting and other risks of bias. The nine criteria are assessed in three categories: low risk (1 point), high risk (0 point) and unclear risk (0 point) and the total quality score ranges from 0 to 9. Two reviewers separately assessed the risk of bias; any disagreements over criteria were discussed until the two reviewers reached a consensus.

## 3 RESULTS

**Figure 1:**
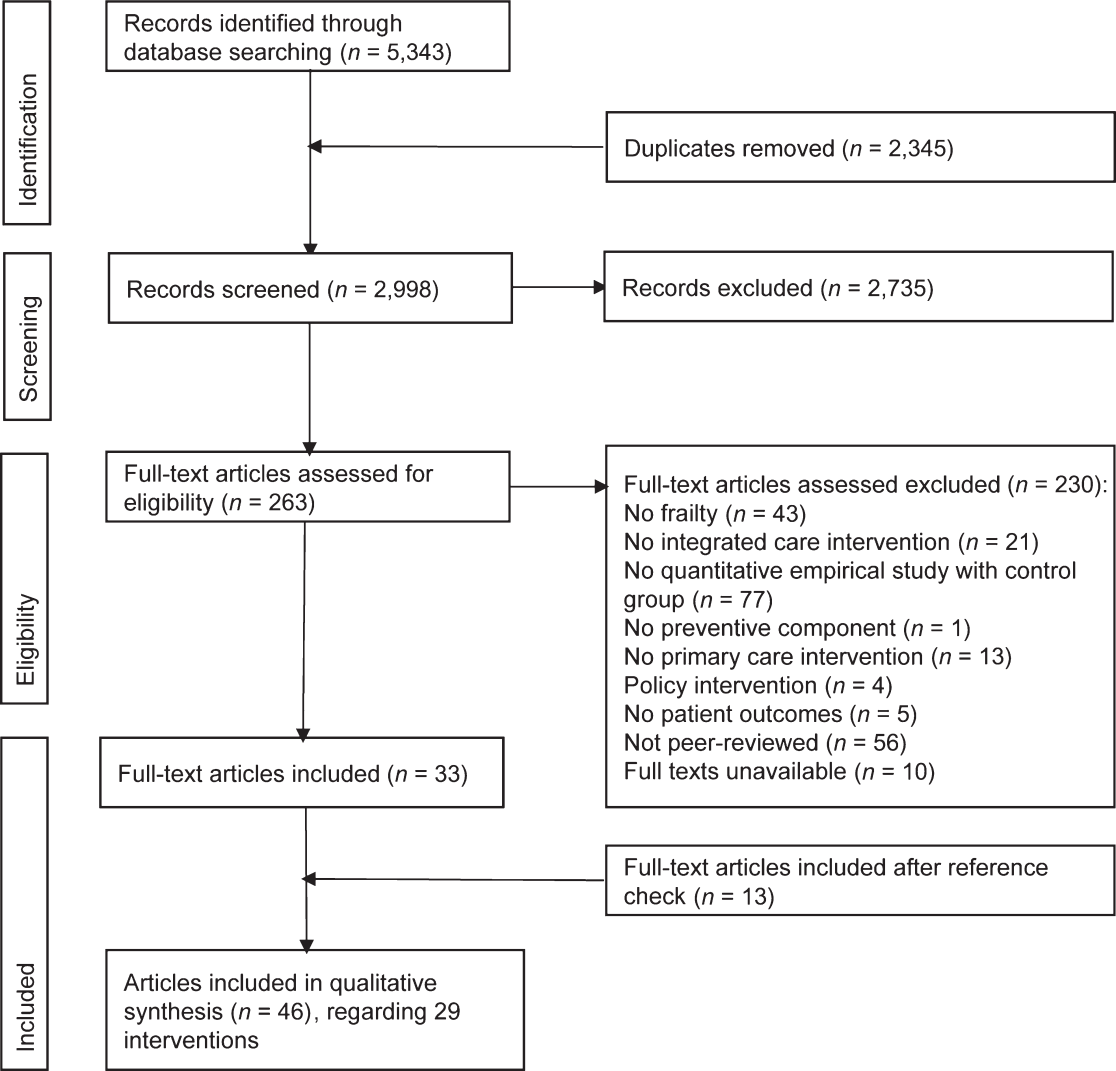
PRISMA flow chart

Figure 1 presents the PRISMA flow chart. Our review included 46 studies regarding a total of 29 separate interventions. The 29 interventions were carried out in 10 countries (see Table 1): Canada (*n* = 8), United States (*n* = 7), the Netherlands (*n* = 6), Sweden (*n* = 2), and Australia, Finland, France, Hong Kong, Japan, New Zealand (*n* = 1 each).

**Table 1:** Study characteristics

Authors	Inclusion criteria participants	Dimension(s) of frailty	Study design	Types of outcomes	N baseline	Follow‐up period (months)	Country	EPOC‐score
Béland et al. (2006)	65+, recruited from community programmes, screened with Functional Autonomy Measurement System (ADL, IADL, communication and cognition), included: score ≤−10	Physical, psychological	Randomised controlled trial	Health, caregiver, process, utilisation	Experimental group *n* = 656; control group (*n* = 653)	22	Canada	6
Bleijenberg et al. (2014)	60+ GP patients, screened by GPs routine care electronic medical record on multimorbidity (frailty index 50) potential health deficits, polypharmacy (chronic use of medication) and consultation gap (no general practice consultation), included: frailty index >0, 20 or ≥5 different medications or consultation gap >3 years	Physical, psychological, social	Cluster‐randomised controlled trial	Health, process, utilisation	Experimental group U‐PRIM (*n* = 790); experimental group U‐PRIM+U‐CARE (*n* = 1,446); control group (*n* = 856)	12	The Netherlands	5
Drubbel et al. (2014)				Cost‐effectiveness	Experimental group U‐PRIM (*n* = 790); experimental group U‐PRIM+U‐CARE (*n* = 1,446); control group (*n* = 856)	12		3
Burns et al. (1995)	65+ admitted to medical, surgical or neurology services at the Veteran Affairs Medical Centre, medical records were screened for following criteria: ≥1 ADL, two or more medical conditions (e.g. congestive heart failure, COPD, diabetes), ≥2 acute care hospitalisations in the previous year or ≥6 scheduled prescription drugs, included: ≥2 criteria	Physical	Randomised controlled trial	Health, utilisation	Experimental group (*n *= 60); control group (*n* = 68)	12	United States	8
Burns et al. (2000)				Health, utilisation	Experimental group (*n* = 60); control group (*n* = 68)	24		5
Dalby et al. (2000)	70+ patients of primary care practice, screened by questionnaire, included: functional impairment or admission to hospital or bereavement in the previous 6 months	Physical, psychological, social	Randomised controlled trial	Health, utilisation	Experimental group (*n *= 73); control group (*n* = 69)	14	Canada	7
De Stampa et al. (2014)	65+, recruited from hospitals or community‐based health services centres, assessed with Contact Assessment Tool (ADL, cognitive deficiency, perceived health, shortness of breath and two social items), included: being very frail with complex health and social needs	Physical, psychological, social	Controlled before‐and‐after study	Health, utilisation	Experimental group (*n* = 105); control group (*n* = 323)	12	France	4
Ekdahl et al. (2016)	75+, identified through administrative healthcare registry, included: ≥3 inpatient hospital care in previous 12 months and ≥3 concomitant medical diagnoses	Physical	Randomised controlled trial	Health, utilisation	Experimental group (*n* = 208); control group (*n* = 174)	36	Sweden	5
Engelhardt et al. (1996)	55+, patients from Veteran Affairs Medical Centre outpatient clinic with ≥10 clinic visits in previous 12 months, screened for functional disability using a standard protocol that assesses level of dependence in the performance of ADL and IADL	Physical	Randomised controlled trial	Health, process, utilisation	Experimental group (*n *= 80); control group (*n* = 80)	16		6
Toseland et al. (1996)				Health, process, utilisation	Experimental group (*n* = 80); control group (*n* = 80)	8	United States	4
O'Donnell and Toseland (1997)				Utilisation	Experimental group (*n* = 80); control group (*n* = 80)	48		5
Fairhall et al. (2015)	70+ patients discharged from Rehabilitation and Aged Services, met Cardiovascular Health Study criteria: weak grip, slow gait, exhaustion, low energy expenditure, and weight loss, inclusion: ≥3 criteria	Physical	Randomised controlled trial	Cost‐effectiveness	Experimental group (*n* = 120); control group (*n* = 121)	12	Australia	6
Gagnon et al. (1999)	70+, patients discharged from Emergency Department in previous 12 months, screened with OARS‐ADL, OARS‐IADL and Boult Assessment tool (probability admission to hospital measuring self‐rated health, admission to hospital in previous 12 months, physician or clinic visit in previous 12 months, every history of cardiac disease, and current availability of caregiver), included: ≥1 OARS‐ADL or ≥2 OARS‐IADL and Boult ≥40%	Physical	Randomised controlled trial	Health, process, utilisation	Experimental group (*n* = 212); control group (*n* = 215)	10	Canada	7
Gray et al. (2010)	50+, screened from family practice Electronic Medical Records for: ≥1 ED visit in previous year, multiple health conditions (including ≥2 chronic conditions for which ≥2 visits were recorded in previous year or 4 conditions with ≥2 visits in previous year), frequent visits (≥5 visits to practice previous 6 months or 10 in previous year), polypharmacy (≥4 currently active or chronic medications), physicians codified the screened patients in 4 risk levels, included: high or medium risk	Physical	Randomised controlled trial	Cost‐effectiveness	Experimental group (*n* = 74); control group (*n* = 78)	12–18	Canada	3
Hébert et al. (2008)	75+, screened for being at risk for functional decline by Sherbrooke Postal Questionnaire (e.g. ≥3 medications and mobility), included: score ≥3	Physical	Controlled before‐and‐after study	Health, caregiver, process, utilisation	Experimental group (*n* = 501); control group (*n* = 419)	12	Canada	2
Hébert et al. (2010)				Health, caregiver, process, utilisation	Experimental group (*n* = 501); control group (*n* = 419)	48		2
Hinkka et al. (2007)	65+ meeting the criteria of entitlement for Pensioners’ Care Allowance—a benefit granted by the SII to compensate for the costs for the person's care at home and is granted to people with a medical disability verified by a physician, and a need of assistance	Physical	Randomised controlled trial	Health, process	Experimental group (*n* = 343); control group (*n* = 365)	12	Finland	7
Kehusmaa et al. (2010)				Cost‐effectiveness	Experimental group (*n* = 376); control group (*n* = 365)	12	Finland	7
Kerse et al. (2014)	75+ patients primary care practice, screened with Brief Risk Identification Tool on health (physical, psychological, cognitive) and disability, included: ≥3	Physical, psychological	Cluster‐randomised controlled trial	Health, process, utilisation	Experimental group (*n* = 1,942); control group (*n* = 1,747)	36	New Zealand	6
Kono et al. (2012)	65+, identified from list of Long‐Term Care Insurance certified residents at the local government office, included: support level 1 or 2 (able to walk, without serious cognitive problems, dependency IADL)	Physical, psychological	Randomised controlled trial	Health, utilisation	Experimental group (*n* = 161); control group (*n* = 162)	24	Japan	5
Kono, Kanaya, Tsumura, and Rubenstein (2013)				Utilisation	Experimental group (*n* = 161); control group (*n* = 162)	24		3
Kono, Izumi, Yoshiyuki, Kanaya, and Rubenstein (2016)				Health, utilisation	Experimental group (*n* = 179); control group (*n* = 181)	36		6
Kristensson, Ekwall, Jakobsson, Midlöv, and Hallberg (2010)	65+, recruited from clinics at university hospital, primary care centres, home care organisations or contact with research group, included: dependent in ≥2 ADL and ≥2 admissions hospital > or ≥4 visits outpatient or primary care	Physical	Randomised controlled trial	Health	Experimental group (*n* = 23); control group (*n* = 23)	3	Sweden	5
Möller, Kristensson, Midlöv, Ekdahl, and Jakobsson (2014)				Health	Experimental group (*n *= 80); control group (*n* = 73)	12		7
Sandberg, Kristensson, Midlöv, and Jakobsson (2015)				Utilisation	Experimental group (*n* = 80); control group (*n* = 73)	12		8
Sandberg, Jakobsson, Midlöv, and Kristensson (2015)				Cost‐effectiveness	Experimental group (*n* = 80); control group (*n* = 73)	12		6
Leung, Lou, Chan, Yung, and Chi (2010)	Elders with moderate to severe functional impairment measured by Minimum Data Set‐Home Care (multiple domains of function, health, social support and service use)	Physical, social	Case–control study	Health	Experimental group (*n* = 78); control group (*n* = 312)	24	Hong Kong	5
Looman, Fabbricotti, and Huijsman (2014)	75+ GP patients, screened by Groningen Frailty Indicator questionnaire (decrease in physical, cognitive, social and psychological functioning), included: score ≥4	Physical, psychological, social	Controlled before‐and‐after study	Health, process, utilisation	Experimental group (*n* = 205); control group (*n* = 212)	3	The Netherlands	4
Makai et al. (2015)				Cost‐effectiveness	Experimental group (*n* =2 05; control group (*n* = 212)	3		3
Looman, Fabbricotti, de Kuyper, and Huijsman (2016)				Health	Experimental group (*n* = 184); control group (*n* = 193)	12		4
Looman, Huijsman, Bouwmans‐Frijters, Stolk, and Fabbricotti (2016)				Cost‐effectiveness	Experimental group (*n* = 184); control group (*n *= 193)	12		4
Melis, van Eijken, et al. (2008)	70+. GP patients, patient has a health problem that was recently presented to the physician by the patient or informal caregiver, request for help is related to the following fields: cognitive disorders, behavioural and psychological symptoms of dementia, mood disorders, mobility disorders and falling or malnutrition, patients/informal caregiver have determined a goal to achieve, fulfil one or more of these criteria: MMSE ≤26, GARS ≥25 or MOS mental health ≤75	Physical, psychological, social	Randomised controlled trial	Health	Experimental group (*n* = 85); control group (*n* = 66)	6	The Netherlands	8
Melis, Adang, et al. (2008)				Cost‐effectiveness	Experimental group (*n* = 85); control group (*n *= 66)	6		6
Metzelthin et al. (2013)	70+ GP patients, screened by Groningen Frailty Indicator questionnaire, score ≥5	Physical, psychological, social	Cluster‐randomised controlled trial	Health	Experimental group (*n* = 193); control group (*n* = 153)	24	The Netherlands	8
Metzelthin et al. (2015)				Cost‐effectiveness	Experimental group (*n* = 193); control group (*n* = 153)	24		5
Montgomery and Fallis (2003)	65+, referred to home care programme. Included: multiple problems requiring coordinated follow‐up home care	Unclear	Randomised controlled trial	Health, caregiver, process, utilisation	Experimental group (*n* = 82); control group (*n* = 82)	3	Canada	3
Morishita et al. (1998)	70+, Medicare beneficiaries, screened with survey on high probability of repeated admissions to hospitals during the following 4 years (e.g. poor self‐related health, previous admission, and morbidity), included: probability ≥40	Physical	Randomised controlled trial	Process	Experimental group (*n* = 248); control group (*n* = 274)	18	United States	3
Boult et al. (2001)				Health, utilisation	Experimental group (*n* = 274); control group (*n* = 294)	18		9
Reuben et al. (1999)	65+ recruited from community‐based sites where older persons congregate, screened with a medical and functional questionnaire (functional status, urinary continence, falls and depression), included: ≥1 warning zones of functional status scale, 2 affirmative answers to both incontinence questions, affirmative answer to the falls screening question and ≥1 supplemental fall question, affirmative answer to the depression question and core Geriatric Depression Scale ≥11	Physical, psychological	Randomised controlled trial	Health, process	Experimental group (*n* = 180); control group (*n* = 183)	15	United States	9
Rockwood et al. (2000)	Patients from rural family practitioners, included: concern about community living, recent bereavement, hospitalisation or acute illness, frequent physician contact, multiple medical problems, polypharmacy, adverse drug events, functional impairment or functional decline, and diagnostic uncertainty	Physical, social	Randomised controlled trial	Health, utilisation	Experimental group (*n* = 95); control group (*n* = 87)	12	Canada	5
Rubenstein et al. (2007)	65+ patients from practice groups with ≥1 visit to ambulatory care centre in previous 18 months, screened by Geriatric Postal Screening Survey (five common geriatric conditions and health‐related symptoms/problems), included: score ≥4	Physical	Randomised controlled trial	Health, utilisation	Experimental group (*n* = 380); control group (*n* = 412)	36	United States	8
Ruikes et al. (2016)	70+ GP patients, identified with EASYcare TOS, a two‐step screening, GP reviewed medical records and answered 14 questions about functioning in somatic, psychological and social domains, structured assessment with EASYcare by practice nurse, included: defined as frail based on clinical reasoning using all explicit and tacit knowledge	Physical, psychological, social	Controlled before‐and‐after study	Health, utilisation	Experimental group (*n* = 287); control group (*n* = 249)	12	The Netherlands	3
Schraeder, Fraser, Clark, and Long (2008)	65+, recruited from primary care practices and hospitals, screened for high risk for mortality, functional decline or health service use with Community Assessment Risk Screen, included: high risk	Physical	Controlled before‐and‐after study	Utilisation	Experimental group (*n* = 400); control group (*n* = 277)	36	United States	3
Shapiro and Taylor (2002)	Older adults referred to waiting list to receive social services by hospitals, rehabilitation centres, physicians, risk score calculated based on uniform state‐wide assessment device based on chronic health conditions, activities of daily living limitations, and other measures of physical and psychological impairment, included: characterised as moderate risk	Physical, psychological	Randomised controlled trial	Health, utilisation	Experimental group (*n* = 40); control group (*n* = 65)	18	United States	6
Tourigny et al. (2004)	75+, recruited from review of files, included: had used home care, day care, or rehabilitation or geriatric ambulatory services previous 12 months AND needed help ≥2 ADL OR needed help ≥1 ADL and one of the following diagnoses: Parkinson's disease, stroke or dementia	Physical	Controlled before‐and‐after study	Health, caregiver, process, utilisation	Experimental group (*n* = 272); control group (*n* = 210)	36	Canada	5
Van Leeuwen et al. (2015)	65+ patients of primary care physician, identified as being frail by physician based on multidimensional definition (experiencing one or more limitations in physical, psychological or social areas) and screened with Program on Research for Integrating Services for the Maintenance of Autonomy Case finding tool (PRISMA‐7), included: score ≥3	Physical, psychological, social	Stepped‐wedge cluster‐randomised controlled trial	Cost‐effectiveness	Group 1 (*n *= 456); group 2 (*n* = 227); group 3 (*n* = 338); group 4 (*n* = 226)	24	The Netherlands	3

Most studies were randomised controlled trials (*n *= 18). Other types were controlled before‐and‐after studies (*n *= 6), cluster‐randomised controlled trials (*n *= 3), case–control study and stepped‐wedge cluster‐randomised controlled trial (*n *= 1 for both). Of the 46 included studies, 36 reported the effectiveness and 10 the cost‐effectiveness of an integrated care intervention. The total number of participants ranged from 36 participants to 3,689 participants. The follow‐up period varied from 3 to 48 months. Overall, the quality of the evidence was moderate ranging from 2 to 9 on the EPOC risk‐of‐bias scale with an average score of 5.3 (see also Table S1).

Our results revealed that each intervention defined frailty differently. All interventions used different tools and inclusion criteria and the dimensions of frailty differed considerably between the interventions. Of the 29 interventions, 13 incorporated the physical dimension of frailty in their inclusion criteria. Five interventions combined the physical and psychological dimensions of frailty and two focused on the physical and social dimension. Eight interventions adopted a broader approach to frailty, including the physical, psychological and social domains of functioning. Additionally, researchers used different age criteria, ranging from 50 years and older to 75 years and older and most interventions adopted the criterion of 65 years and older.

### 3.1 Interventions

The 29 interventions were arranged according to the Valentijn framework (Valentijn et al., 2013; see Table S2). The level of integration of the interventions is high at the micro level but generally low at the meso and macro levels of integration.

*Service integration* was substantially high in all 29 interventions. All interventions used assessment tools, mostly a comprehensive geriatric assessment, which the majority of interventions used to develop a care plan. Occasionally, the frail older person and their informal caregiver were also involved in the development of the care plan. The assessments and care plans revealed the preventive character of the integrated interventions. The assessment demonstrated that it could detect a wide range of problems that might not have been recognised in usual care. The care plan addressed a selection of these problems; however, the articles provided limited insight into how the assessments resulted in a care plan.

Despite the similarities in assessments and care plans, the follow‐up differed between interventions, particularly in the role of prevention. Predominantly, case management was an important part of the follow‐up which involved executing the care plan, monitoring the frail older people, advocacy by arranging admission to services and updating other professionals. Follow‐up could also include home visits or specific interventions aimed at fall prevention or activation. Follow‐up standardisation fluctuated: some interventions developed protocols so that follow‐up took place each month, whereas other interventions were more flexible, responding to the needs of the frail older people. Remarkably, the role of prevention in the follow‐up was generally limited and differed between interventions. A few interventions (*n* = 9) paid explicit attention to health education, health promotion or adopting an active lifestyle and coping.

*Professional integration* varied between interventions. Different professionals were responsible for follow‐up: (practice) nurses, social workers, physiotherapists, geriatricians or a multidisciplinary team of professionals. The involved professionals and organisations differed between interventions. Physicians and nurses are involved most frequently but also collaboration with geriatricians in secondary care and social workers commonly occurs (both *n* = 13). Some interventions were situated in a clear focal organisation, such as a primary care or community practice, home‐care organisation, Geriatric Evaluation and Management outpatient clinic, physiotherapist or rehabilitation centre, whereas other interventions are situated in a network of organisations. The level of involvement of the GP varied between the interventions; the GP was at the core of some of the interventions, whereas occasionally the GP had no role at all and the integrated care intervention co‐existed alongside usual care. Finally, the intervention‐specific education of professionals was sparse and concentrated mostly on very specific elements of the interventions such as assessment instruments or protocols.

*Organisational integration* was modest in the preventive, integrated care interventions. A few cases created a network of organisations: five cases set up a Joint Governing Board and two built a new consortium. *Financial integration* was even less frequent. Two interventions had partial financial integration; one was fully integrated financially and its teams controlled their own budget.

*Functional integration* was limited; a few interventions (*n* = 9) used a shared information system or developed multidisciplinary protocols (*n* = 6) on specific themes such as urinary incontinence or falls. In addition, the level of *normative integration* was negligible (*n* = 4) according to the intervention descriptions. Workshops and training courses focused on the following topics: collaboration of the practice nurse and GP; goals and responsibilities of collaborative care teams; team development; client‐centredness and interdisciplinary collaboration.

Informal caregivers of the frail older people were not always considered as active participants by the professionals in the interventions. Sporadically (*n* = 2), the caregiver burden was included in the comprehensive assessment and occasionally (*n* = 6) the follow‐up was also aimed at the informal caregivers. At times (*n* = 5), the professionals actively involved informal caregivers in the care process, by validating the care plan with them or involving them in the actual decision‐making process.

### 3.2 Health outcomes

There was generally limited evidence of integrated care interventions on health outcomes of frail older people. No clear pattern emerged in the elements or level of integration of the interventions that did generate significant effects.

An extensive range of health outcomes were considered (see Table 2). The outcomes reported most often were activities of daily living (ADL)/instrumental activities of daily living (IADL) (*n* = 18), mortality (*n* = 15) and physical functioning (*n* = 13). Less frequently used outcomes were social support (*n* = 3), vitality (*n* = 3) and desire for institutionalisation and frailty (*n* = 1 for both).

**Table 2:** Health outcomes

Authors	Perceived health	Morbidities	ADL/IADL	Physical functioning	Pain	Vitality	Mental health	Depression	Role	Social functioning	Social support	Cognitive functioning	QoL—general	Qol—health‐related	QoL—well‐being	Life satisfaction	Fall incident	Mortality	Desire institutionalisation	Frailty
Béland et al. (2006)	0																	0		
Bleijenberg et al. (2014)			+	0		0	0			0			0	0				0		
Drubbel et al. (2014)														0						
Burns et al. (1995)	+	−	0	0				+		+		0				+		0		
Burns et al. (2000)	+		0/+					+		+		+			+	+		0		
Dalby et al. (2000)																		0		
de Stampa et al. (2014)		+	0		0			+		0		0					0	0		
Ekdahl et al. (2016)																		+		
Engelhardt et al. (1996)	0			0			0	0			0				0			+		
Toseland et al. (1996)	0			0			0	0	0						0			+		
O'Donnell and Toseland (1997)																				
Fairhall et al. (2015)														0						+
Gagnon et al. (1999)	0		0	0	0	0	0		0	0			0							
Gray et al. (2010)																				
Hébert et al. (2008)			0									+								
Hébert et al. (2010)			+																	
Hinkka et al. (2007)	+	0		0	0							0								
Kehusmaa et al. (2010)				0										0						
Kerse et al. (2014)			0	+				0					+							
Kono et al. (2012)			0					0			0									
Kono et al. (2013)																				
Kono et al. (2016)			+/0					0				0				−	0	0		
Kristenson et al. (2010)								0						0						
Möller et al. (2014)			0/	0													0			
Sandberg, Kristensson et al. (2015)																				
Sandberg, Jakobsson, et al. (2015)																				
Leung et al. (2010)																	+			
Looman et al. (2014)	0		0				0			0			0	0	+					
Makai et al. (2015)														0	0					
Looman, Fabbricotti, et al. (2016)	0		0				0			0			+	0	+					
Looman, Huijsman, et al. (2016)														0						
Melis, van Eijken, et al. (2008)			+	0			+		0	0		0	0		+			0		
Melis, Adang, et al. (2008)			0				0													
Metzelthin et al. (2013)			0					0		0	0						0			
Metzelthin et al. (2015)			0											0						
Montgomery and Fallis (2003)			0	0								+								
Morishita et al. (1998)																				
Boult et al. (2001)				+				+										0		
Reuben et al. (1999)	0			+	0	+	0		0	+					0			0		
Rockwood et al. (2000)			0	0								0	0					0		
Rubenstein et al. (2007)	0	0	0					0									0			
Ruikes et al. (2015)			0				0			0				0				0		
Schreader et al. (2008)																				
Shapiro and Taylor (2002)								+		+					+	+		+		
Tourigny et al. (2004)			+															0	+	
van Leeuwen et al. (2015)			0	0			0							0						

+: significant outcome in favour of the intervention; 0: no significant outcome; −: significant outcome in favour of the control group.

In terms of effectiveness, four outcomes were most promising: well‐being, life satisfaction, frailty and desire for institutionalisation. The majority of the interventions reporting these specific outcomes found a positive effect for the intervention. However, these outcomes were reported less frequently, especially desire for institutionalisation and frailty. For other outcomes, positive effects were reported occasionally; for instance, depression (*n* = 4 out of 10) and cognitive functioning (*n* = 3 out of 8). Four outcome measures did not reach significance in any of the interventions: pain, role, social support and health‐related quality of life. We found an effect in favour of the control group only twice: reported morbidities (Burns, Nichols, Graney, & Cloar, 1995) and life satisfaction (Kono et al., 2016).

The differences in outcomes could not be explained by the elements and level of integration of the interventions. This, for example, is shown by the 18 interventions that reported ADL and IADL as an outcome. Four interventions that showed positive effects had, for example, a multidisciplinary team, whereas the two other interventions with positive effects had no multidisciplinary team. The same mixed pattern was found in the 12 interventions that reported no effects on ADL and IADL. Some outcomes tended to show that better outcomes were accompanied by a lower level of integration. The studies that showed an effect on mortality in favour of the intervention were not integrated normatively, organisationally or financially. The interventions that reported a positive effect on mental health were not integrated functionally, normatively or organisationally.

Two remarkable effective interventions showed similar effects for life satisfaction, well‐being, depression and social functioning. One intervention (Shapiro & Taylor, 2002) also found significant effects in mortality, whereas the other also reported effects on perceived health, cognitive functioning and IADL (Burns, Nichols, Martindale‐Adams, & Graney, 2000; Burns et al., 1995). These results highlighted the limited effect in the physical domain of functioning. Both these interventions showed a low level of integration at the meso and macro level since both had no functional, organisational and financial integration.

### 3.3 Outcomes for informal caregivers and professionals

Our results show a considerable lack of emphasis on outcomes regarding the informal caregivers and professionals. Subsequently, the effects on these outcomes were negligible.

Nine of the 29 interventions reported on the following outcomes: caregiver's satisfaction with care, caregiver's desire for institutionalisation, caregiver's subjective and objective burden and professional satisfaction with care (Table 3). The effect on caregiver's satisfaction with care was most convincing, since it was effective in one of the two studies reporting this outcome. Caregiver's satisfaction improved in an intervention which encouraged family participation in care and decision‐making and professionals also intervened with caregivers (Beland et al., 2006). No effect was found in another intervention where no specific attention was paid to the informal caregiver (Montgomery & Fallis, 2003). Caregiver's desire for institutionalisation did not show any significant effect.

**Table 3:** Outcomes for informal caregivers and professionals

	Caregiver burden—subjective	Caregiver burden—objective	Caregiver desire for institutionalisation	Caregiver satisfaction	Professional satisfaction
Béland et al. (2006)	0			+	
Bleijenberg et al. (2014)					
Drubbel et al. 2014		NS			
Burns et al. (1995)					
Burns et al. (2000					
Dalby et al. (2000)					
de Stampa et al. (2014)					
Ekdahl et al. (2016)					
Engelhardt et al. (1996)					
Toseland et al. (1996)					
O'Donnell and Toseland (1997)					
Fairhall et al. (2015)					
Gagnon et al. (1999					
Gray et al. (2010)					
Hébert et al. (2008)	0		0		
Hébert et al. (2010)	‐		0		
Hinkka et al. (2007)					
Kehusmaa et al. (2010					
Kerse et al. (2014)					
Kono et al. (2012)					
Kono et al. (2013)					
Kono et al. (2016)					
Kristenson et al. (2010)					
Möller et al. (2014)					
Sandberg, Kristensson et al. (2015)					
Sandberg, Jakobsson, et al. (2015)		+			
Leung et al. (2010)					
Looman et al. (2014)					
Makai et al. (2015)		0			
Looman, Fabbricotti, et al. (2016)					
Looman, Huijsman, et al. (2016)		0			
Melis, van Eijken, et al. (2008)					
Melis, Adang, et al. (2008)					
Metzelthin et al. (2013)					
Metzelthin et al. (2015)		0			
Montgomery and Fallis (2003)	0			0	
Morishita et al. (1998)					NS
Boult et al. (2001)					
Reuben et al. (1999)					
Rockwood et al. (2000)					
Rubenstein et al. (2007)					
Ruikes et al. (2015)					
Schreader et al. (2008)					
Shapiro and Taylor (2002)					
Tourigny et al. (2004)	+				
van Leeuwen et al. (2015)		0			

+: significant outcome in favour of the intervention; 0: no significant outcome; −: significant outcome in favour of the control group; NS: outcome not tested for significance.

The effects on caregiver subjective burden were rather inconsistent. Four studies reported this outcome, all using the same measurement instrument, but the results were mixed: an effect in favour of the intervention (Tourigny, Durand, Bonin, Hebert, & Rochette, 2004), the control group (Hébert et al., 2010) or no effect at all (Béland et al., 2006; Montgomery & Fallis, 2003). These results were unrelated to the role of the informal caregiver in the intervention since informal caregivers were the least involved in the care process in the most effective intervention. The objective burden of informal caregivers was not affected by preventive, integrated care interventions. The objective burden—time spent on informal care—was considered from a societal perspective in five cost‐effectiveness analyses and one intervention found an effect in favour of the caregivers in the intervention group. Time spent on IADL by the caregivers decreased in this intervention that aimed specially at improving the functional status of frail older people (Sandberg et al. 2015).

Professional satisfaction was the only outcome regarding professionals that was taken into account by a single study (Morishita, Boult, Boult, Smith, & Pacala, 1998). However, this study did not apply significance testing. The professionals indicated that the intervention is appropriate, helpful for both their patients and themselves in ongoing care for their patients.

### 3.4 Process outcomes

Process outcomes of integrated care interventions generated little interest but the effects were beneficial, particularly for care process. Five types of outcomes fit into the category of process outcomes: goal attainment, empowerment, satisfaction with care, care process and rate of implementation (Table 4).

**Table 4:** Process outcomes

Authors	Goal attainment	Empowerment	Satisfaction with care	Care process	Implementation
Béland et al. (2006)			0		
Bleijenberg et al. (2014)			0		
Drubbel et al. (2014)					
Burns et al. (1995)					
Burns et al. (2000)					
Dalby et al. (2000)					
de Stampa et al. (2014)					
Ekdahl et al. (2016)					
Engelhardt et al. (1996)			+	+	
Toseland et al. (1996)			+	+	
O'Donnell, Toseland 1997)					
Fairhall et al. (2015)					
Gagnon et al. (1999)			0		
Gray et al. (2010)				+	
Hébert et al. (2008)		0	0		NS
Hébert et al. (2010)		+	+		NS
Hinkka et al. (2007)			NS		
Kehusmaa et al. (2010)					
Kerse et al. (2014)			0		
Kono et al. (2012)					NS
Kono et al. (2013)					
Kono et al. (2016)		0		+	
Kristenson et al. (2010)					
Möller et al. (2014)					
Sandberg, Kristensson, et al. (2015)					NS
Sandberg, Jakobsson, et al. (2015)					
Leung et al. (2010)					
Looman et al. (2014)			0		
Makai et al. (2015)					
Looman, Fabbricotti, et al. (2016)					
Looman, Huijsman, et al. (2016)					
Melis, van Eijken, et al. (2008)					
Melis, Adang, et al. (2008)					
Metzelthin et al. (2013)					NS
Metzelthin et al. (2015)					
Montgomery and Fallis (2003)				+	
Morishita et al. (1998)			+		
Boult et al. (2001)					
Reuben et al. (1999)		0	0		
Rockwood et al. (2000)	+				NS
Rubenstein et al. (2007				+	
Ruikes et al. (2015)					
Schreader et al. (2008)					
Shapiro and Taylor (2002)		+			
Tourigny et al. (2004)					
van Leeuwen et al. (2015)					

+: significant outcome in favour of the intervention; 0: no significant outcome; −: significant outcome in favour of the control group; NS: outcome not tested for significance.

For three types of outcomes, most effects were in favour of the intervention group: goal attainment, empowerment and care process. Goal attainment was reported for only one intervention as the primary outcome measure (Rockwood et al., 2000), in which an effect in favour of the intervention was generated. Empowerment had a positive effect in two of four interventions. The definition of empowerment was aligned with the focus of intervention studies: it was related either to patient involvement in the care process or to empowerment in terms of activities of daily life. Both definitions showed a significant effect once.

The care process improved in all five integrated, preventive care interventions in which it was considered an outcome measure. These five interventions were not integrated normatively, organisationally or financially. The operationalisation of care process differed between studies and was closely aligned to specific interventions. For example, the Rubenstein intervention focused on five geriatric target conditions and referrals. The researchers operationalised the care process by evaluating documentation and assessing the target conditions and referrals (Rubenstein et al., 2007).

Evidence for the most common outcome in this category—satisfaction with care—was not convincing. Of the 10 interventions reporting on this outcome, three found an increase in satisfaction with preventive, integrated care. No clear pattern emerged on what could explain the differences in effects. Two Outpatient Geriatric Evaluation Management interventions in the United States reported higher satisfaction with care (Engelhardt, Toseland, & O'Donnell, 1996; Morishita et al., 1998; Toseland, O'Donnell, & Engelhardt, 1996) but a very comparable intervention, also using a similar measurement instrument, did not result in higher satisfaction (Reuben, Frank, Hirsch, McGuigan, & Maly, 1999). PRISMA resulted in higher satisfaction with care after 4 years (Hébert et al., 2010) but this effect was not yet established after 1 year (Hébert, Dubois, Raiche, Dubuc, & Group, 2008). Comparable interventions to PRISMA with a high level of professional integration (Kerse et al., 2014) and organisational integration (Béland et al., 2006; Gagnon, Schein, McVey, & Bergman, 1999; Looman et al. 2014) found no effect in shorter follow‐up periods (3–36 months).

### 3.5 Healthcare utilisation

Healthcare utilisation did not differ substantially between frail older people receiving care as usual and preventive, integrated care. Nonetheless, we observed both decreases and increases in utilisation.

Healthcare utilisation was the most reported outcome (*n* = 27; Table 5). The focus was mainly on secondary care since the most frequently reported outcomes were hospital length of stay (*n* = 19), hospital admission (*n* = 18) and nursing home admission (*n* = 18). Far less attention was paid to social care utilisation such as psychosocial care (*n* = 4) or meals on wheels (*n* = 5). The least reported outcomes were diagnostics (*n* = 4) and equipment (*n* = 3).

**Table 5:** Healthcare utilisation

Authors	GP/primary care	Contact physicians outpatient care	Paramedical care	Home care	Day care	Diagnostics	Equipment	Meals on wheels	Psychosocial care	Hospital admission	Hospital length of stay	Emergency department	Day surgery	Nursing home	Medication	Costs
Béland et al. (2006)				−						+		0		0		0
Bleijenberg et al. (2014)	−									0		0				
Drubbel et al. (2014)	NS			NS	NS						NS	NS		NS		0
Burns et al. (1995)										0					+	
Burns et al. (2000)	+									0						
Dalby et al. (2000)	0	0								0	0	0			‐	
de Stampa et al. (2014)										+/−		+				
Ekdahl et al. (2016)	−		−	0		0	0			0	+			0	0	0
Engelhardt et al. (1996)	−					0				0	0	+	0	0	0	0
Toseland et al. (1996	−	0				0				0	0	0	0	0		0
O'Donnell, Toseland (1997)	−					0			0	0		0	0			
Fairhall et al. (2015)	0			0				−	0		0			0		
Gagnon et al. (1999)										0	0	−				
Gray et al. (2010)	0		0			‐					0	0	0		0	−
Hébert et al. (2008)	‐	0	‐	‐	‐			‐	0	0	−	−/+	0	0		
Hébert et al. (2010)	0	0	0	0	0				0	0	0	−/+	0	0		
Hinkka et al. (2007)																
Kehusmaa et al. (2010	0	0		0		0		0			0		0	0	0	‐
Kerse et al. (2014)		0	0	+						0		0		−		
Kono et al. (2012)				−										−		0
Kono et al. (2013)																+
Kono et al. (2016)				0						0				0		0
Kristenson et al. (2010)																
Möller et al. (2014)																
Sandberg, Kristensson et al. (2015)		+								0	0	+				
Sandberg, Jakobsson, et al. (2015)		0		0							0					0
Leung et al. (2010)																
Looman et al. (2014)			0	0	0			0	0							
Makai et al. (2014)	−	0	0	0	0				0		0	0	0	0		0
Looman, Fabbricotti, et al. (2016)																
Looman, Huijsman, et al. (2016)	−		0	0	0				0		0			0		0
Melis, van Eijken, et al. (2008)																
Melis, Adang, et al. (2008)	0	0	0	0	0			0			0			0		0
Metzelthin et al. (2013)																
Metzelthin et al. (2015)	−	0	−	0			0				0			0		0
Montgomery and Fallis (2003)				−	−					0	+			+	−	
Morishita et al. (1998)																
Boult et al. (2001)		0		−			0				0			0		0
Reuben et al. (1999)																
Rockwood et al. (2000)														0		
Rubenstein et al. (2007)										0	0					
Ruikes et al. (2015)										0				0		
Schreader et al. (2008)										+	+	0				0
Shapiro and Taylor (2002)														+		
Tourigny et al. (2004)	−/+									0	−	−			0	
van Leeuwen et al. (2015)	0		NS	NS					NS	0	NS		NS	NS	0	0

+: significant outcome in favour of the intervention (i.e. decrease in healthcare utilisation); 0: no significant outcome; −: significant outcome in favour of the control group (i.e. increase in healthcare utilisation); +/− significant outcome both in favour of the intervention and the control group within one category (i.e. both decrease and increase in healthcare utilisation within one category); NS: outcome not tested for significance.

The majority of the interventions reported no significant increase or decrease in healthcare utilisation in any outcome category. Despite the limited effects, some patterns in healthcare utilisation could be revealed. Three types of healthcare utilisation were not affected at all by integrated care: use of equipment, psychosocial care and day surgery. The effects of integrated care interventions on hospital care tend to be positive; slightly more interventions showed a decrease in hospital care utilisation by the frail older people than an increase. This accounted for four types of hospital care: admission to the emergency department, length of stay in hospital, admission to the hospital and contact with physicians in outpatient care. On the other hand, more increases than decreases in utilisation were reported for other types of care. Primary care increased for almost half of the interventions reporting this outcome. For paramedical care, day care, diagnostics and meals on wheels only increases in utilisation were observed, although led by non‐significant effects for all types of healthcare utilisation. The effect on nursing home admissions was ambiguous since 14 interventions found no effects, two showed a decrease in admissions (Montgomery & Fallis, 2003; Shapiro & Taylor, 2002) and two an increase (Kerse et al., 2014; Kono et al., 2012). In 14 interventions, the healthcare utilisation outcomes were converted into costs. The effects were sparse; 11 interventions find no significant effect, due mostly to the wide variation in costs.

At intervention level, six interventions reported no significant effects at all for healthcare utilisation. Moreover, a substantial number (*n* = 12) of interventions reported more increases in healthcare utilisation than decreases. Remarkably, the PRISMA intervention reported increases in six types of healthcare utilisation in the first year of follow‐up (Hébert et al., 2008), but these increases disappeared (i.e. became non‐significant) in the 4‐year follow‐up period (Hébert et al., 2010).

The differences in outcomes in healthcare utilisation could not be fully explained by the differences in components or level of integration of the interventions. The results indicated that a higher level of integration did not result in better outcomes. For instance, for hospital length of stay, there was no organisational and financial integration in the interventions that generated a decrease in length of stay, whereas the interventions that had an increase in length of stay were integrated organisationally and financially. The one intervention that resulted in a decrease in primary care had no functional, organisational and financial integration, whereas this was both present and absent for interventions that found no effect or an increase in primary care utilisation.

### 3.6 Cost‐effectiveness

Our systematic review showed limited evidence for the cost‐effectiveness of preventive, integrated care interventions for frail older people. Cost‐effectiveness was determined for nine interventions, of which three stated they were cost‐effective (Table 6). Generally, we observed no significant differences in total cost between the preventive, integrated care interventions and care as usual. The total costs of two interventions were higher than care as usual (Gray, Armstrong, Dahrouge, Hogg, & Zhang, 2010; Kehusmaa, Autti‐Rämö, Valaste, Hinkka, & Rissanen, 2010) due mostly to high intervention costs rather than any increase in healthcare utilisation.

**Table 6:** Cost‐effectiveness

Authors	Perspective	Costs	Effect measure	Effects	Cost‐effective
Drubbel et al. (2014	Societal	0	QALY	0	Yes—95% WTP €20,000
Fairhall et al. (2015)	Healthcare funder	0	Frailty; QALY	+/0	Yes—80% WTP AU $50,000
Gray et al. (2010)	Provincial ministry of health	−	Quality of care	+	No
Kehusmaa et al. (2010)	Societal	−	Functional independence; health‐related quality of life	0	No
Makai et al. (2015)	Societal	0	QALY; ICECAP	0	No
Looman, Huijsman, et al. (2016)	Societal	0	QALY	0	No
Melis, Adang, et al. (2008)	Healthcare system	0	% successful treatment	0	Yes—75% WTP €34,000
Metzelthin et al. (2015)	Societal	0	Disability; QALY	0	No
Sandberg, Jakobsson, et al. (2015)	Societal	0	QALY	0	No
van Leeuwen et al. (2015)	Societal	0	ADL & IADL; physical health; mental health; QALY	0	No

+: significant outcome in favour of the intervention, 0: no significant outcome; −: significant outcome in favour of the control group.

Besides the limited cost savings, the effects of the interventions were also modest, particularly in terms of quality‐adjusted life years (QALY). Seven studies chose QALY as an effect measure and one study adopted another measure for health‐related quality of life. None of these interventions found an effect in favour of the intervention. Two significant effects were established: quality of care for APTcare (Gray et al., 2010) and frailty for FIT (Fairhall et al., 2015). These effect measures were more properly aligned to the two interventions. APTcare, for instance, was a disease management programme and quality of care was determined by specific performance measures for each chronic disease. FIT strongly focused on frailty by assessing specific frailty characteristics and implementing specific interventions for each frailty condition.

Due to their modest effects, the majority of interventions were not cost‐effective. Three interventions had a high probability of being cost‐effective, 75% at a willingness to pay 20,000 euro (Drubbel et al., 2014), 95% at 34,000 euros (Melis et al., 2008) and 80% at 50,000 dollars (Fairhall et al., 2015). These three interventions had some features in common: the absence of case management, a single entry point, information system and organisational and financial integration. These elements were both present and absent in the seven interventions that were not cost‐effective.

## 4 DISCUSSION

The widespread interest in preventive, integrated care has generated high expectations for improving the organisation of care for community‐dwelling frail older people. The aim of this study was to systematically review the empirical evidence for its effectiveness and cost‐effectiveness to test these expectations. Our results showed that the fragmented evidence is not compelling.

Preventive, integrated care is not likely to be effective since the majority of the reported outcomes show no effect. Less frequently reported outcomes were most promising such as care process, well‐being and life satisfaction, even as outcomes closely aligned to the aim of the interventions such as frailty and fall prevention. However, when interventions were specifically aimed at ADL, IADL and physical functioning, effects were less likely to be substantiated. The evidence for healthcare utilisation was mixed but preventive, integrated care did not lead to clear cost reductions or substitution of healthcare and cost‐effectiveness was limited. Our review showed no clear relation between (cost‐) effectiveness and specific preventive, integrated elements or levels of integration. The more integrated interventions, in particular, in terms of functional, normative, organisational and financial integration, tended not to result in more effectiveness. Differences in outcomes could neither be explained by the quality of the studies, the sample size, nor the follow‐up period.

Another important result of our systematic review was that populations, interventions and outcomes differed substantially which made it extremely difficult to compare both interventions and evaluation studies. First, fragmentation was caused by the heterogeneity of the target population of the interventions. No consensus existed on the definition of frailty since the inclusion criteria of the participants were formulated differently in literally all studies. Frailty was mostly related to the physical domain of functioning, but the psychological and social domain were gradually incorporated as well. In the inclusion criteria, the physical domain was very frequently translated to dependency in ADL or IADL, whereas previous research has shown that frailty is a different condition than disability (Fried, Ferrucci, Darer, Williamson, & Anderson, 2004; Lutomski et al., 2014). Second, the interventions were built up differently in terms of elements and level of integration. Some common elements could be derived, such as assessments and care plans but their follow‐up varied between interventions and was not clearly described in the intervention descriptions. Also the role of prevention differed between interventions. Secondary prevention was part of all interventions due to the comprehensive geriatric assessment and care plans. Nevertheless, screening the older population for frailty was less common. Only few interventions paid explicit attention to self‐management, health education and empowerment in the follow‐up of frail older people; thus tertiary prevention was limited. Besides the differences in the elements, the level of integration of the interventions also varied. Some were organisationally integrated interventions but were not normatively and functionally integrated and vice versa. Third, the fragmentation of the evaluation research is caused predominantly by the extensive variation in outcome measures. Some main categories that nearly always are considered to determine the (cost‐)effectiveness of preventive integrated care can be distinguished: ADL and IADL, hospitalisation and nursing home admission. But besides these commonalities, the outcomes were dispersed, ranging from vitality to desire for institutionalisation for frail older people and caregivers. Many different measurement instruments were used for these outcomes which fragmented the evidence even more and made comparisons more difficult. Although measurement of healthcare utilisation was consistent ‐ by self report or from registrations ‐ the outcomes typically focused on healthcare rather than social care and were distinctive for each intervention. These differences also implied that the cost of preventive, integrated care was calculated differently for each intervention.

### 4.1 Interpretation of results in the context of other studies

Our results added nuances to the high expectations for integrated care in the literature. Some theoretical studies on (general) integrated care state that it could pursue a wide range of aims (Kodner & Spreeuwenberg, 2002). However, our results were in line with other empirical reviews on integrated care interventions for older people. Previous research also emphasised the unconvincing effects on health outcomes (Eklund & Wilhelmson, 2009; Johri et al., 2003; Low, Yap, & Brodaty, 2011; Stokes et al., 2015; You et al., 2012). The positive effect on well‐being was confirmed in a systematic review on case management of frail older people and people with dementia (You et al., 2012). Our results confirmed the lack of emphasis on informal caregivers and professionals, in particular (Eklund & Wilhelmson, 2009; Johri et al., 2003; Stokes et al., 2015; You et al., 2012). Previous research showed similar results for the care process but this outcome was considered far less often than health outcomes and healthcare utilisation. Integrated care for patients with chronic diseases also resulted in improvement of the quality of care (Ouwens et al., 2005) and case management for older people resulted in fewer unmet service needs (You et al., 2012). However, our review did not show encouraging effects on care satisfaction, in contrast to case management interventions (Stokes et al., 2015). Our results mitigate the effects of integrated care on healthcare utilisation. Two previous reviews showed a decrease in hospitalisation and institutionalisation (Eklund & Wilhelmson, 2009; Johri et al., 2003). Our results were less conclusive when more types of health and social care utilisation were considered. Indeed, there was an indication that hospital care might decrease because of integrated care intervention but the effect on institutionalisation was inconsistent in our review. Our broader range of outcomes also showed increases in healthcare utilisation, mostly for primary care.

### 4.2 Strengths and limitations

The strength of this systematic review is the comprehensive overview it provides in terms of both interventions and outcomes. Analysing the interventions with the Valentijn theoretical framework with an additional focus on prevention provided useful insights into the various components of integrated care and the different levels of integration in relation to the wide range of outcomes. Besides the included articles, we also considered corresponding study protocols in order to provide all available information on the interventions. Furthermore, we considered all types of outcomes, divided into five categories, one of which was cost‐effectiveness for which systematic evidence is scarce (Ouwens et al., 2005; Stokes et al., 2015).

The first limitation of our systematic review is that we did not perform a meta‐analysis. We were not able to do a meta‐analysis because of the substantial differences in population, interventions, research designs and the wide range of outcomes measured with different instruments. Our aim was to present the bigger picture rather than limiting ourselves to a selection of more common outcome categories. The most common outcomes were ADL/IADL, physical functioning, mortality, hospital admissions, home care and institutionalisation. However, this would have been too restricted to fully explore the potential effectiveness of preventive, integrated care. Our research showed that effects can be observed in other outcomes, such as care process or well‐being.

In providing this broad overview, we had to categorise the outcome measures, which is the second limitation of our study. Many different operationalisations of outcomes could be distinguished, especially for ADL/IADL, physical functioning, hospital admissions and well‐being. A concrete example is the category of hospitalisation that not only includes actual hospitalisation, but also the number of multiple, acute, subacute, planned and total hospitalisations. Another example was physical functioning, for which the following measurements were used in a single intervention: physical functioning, number of restricted activities days, number of bed days, physical performance test, NIA battery score and physical health summary scale (Reuben et al., 1999). In these cases, we adopted an optimistic approach; if one of the outcomes within a category had a positive effect, we reported it as a positive outcome for that category.

The last limitation is the moderate state of empirical evidence, risk of bias and quality of the studies. This was partly due to our inclusion criterion on controlled designs, which implied that non‐randomised trials were also included and that increased the risk of bias. Yet, a more important contributor to the moderate risk of bias was the lack of information in the evaluation studies. The number of EPOC criteria we determined as “unclear risk” was approximately equivalent to the number of criteria determined as “high risk.”

### 4.3 Implications for research, policy and practice

The first implication is that the heterogeneity of frail older people in the community should be further explored. The population of the interventions differed substantially between and within interventions. Several studies adopted a narrow definition of frailty, focusing on the physical domain, but more recent studies also considered the psychological and social domain. Still, there is no consensus on the definition and measurement of frailty (Dent, Kowal, & Hoogendijk, 2016) and thereby on identifying which community‐dwelling older people would benefit most from the preventive, integrated care interventions (Collard, Boter, Schoevers, & Oude Voshaar, 2012). Researchers have become increasingly aware of the complexity and heterogeneity of frailty (see also Eklund & Wilhelmson, 2009) and recently, have distinguished subpopulations of physically frail older people (Lafortune, Béland, Bergman, & Ankri, 2009; Liu, 2014). These subpopulations could further unravel frailty and support professionals in daily practice. However, in evaluations of studies into preventive, integrated care, the population of frail older people is still considered as a single group and no distinction is made between the characteristics of the frail older people. When the population of the intervention is more heterogeneous, it might be harder to achieve effectiveness (Almeida Mello et al., 2016; Ferrucci et al., 2004; Lette, Baan, van den Berg, & de Bruin, 2015). Accordingly, a possible explanation for the limited effectiveness of integrated care might be that it is more beneficial for certain subpopulations of frail older people; this hypothesis should be explored further.

The second implication is that further research should provide better insight into the term “effectiveness” for community‐dwelling frail older people before extensive (expensive) preventive, integrated care interventions are designed, implemented and evaluated. It is crucial to explore what specific outcomes can be influenced for the frail older people—who are deteriorating in multiple domains of functioning—and their informal caregivers. Likewise, it is fundamental to formulate realistic expectations for what preventive, integrated care can achieve. Our systematic review challenges the important role that physical domain of functioning plays in preventive, integrated care for frail older people and its evaluation research. Many professionals involved in integrated care aim specifically at improving ADL/IADL or at preventing functional decline with limited effectiveness. An important question for practice, policy and research is whether we can expect a positive effect for ADL/IADL in preventive, integrated care at all. In fact, a recent systematic review proved that it is very difficult to influence ADL limitations for the older population (van Vorst et al., 2016). The QALY is another outcome that might be less suitable for determining cost‐effectiveness for the community‐dwelling frail older population. This outcome is widely used in the curative sector and is known for its comparability across populations and interventions (Drummond, Sculpher, Claxton, Stoddart, & Torrance, 2005). None of the interventions found an effect on health‐related quality of life and previous research has also confirmed that it might be less appropriate for frail older people (Comans, Peel, Gray, & Scuffham, 2013; Makai, 2014). Our systematic review provides useful support for a shift from (psychical) functioning to well‐being in preventive, integrated care and, correspondingly, its evaluation research. Also well‐being of informal caregivers should be considered since the role of informal caregivers has become more prominent in the care for frail older people (Grootegoed & Van Dijk, 2012). Primary care professionals are originally trained to adopt a monodisciplinary, disease‐specific approach (Lette et al., 2015) but preventive, integrated care requires a more holistic approach including an important role for well‐being (Schuurmans, 2004; Valentijn et al., 2013). Previous research has shown dimensions of well‐being for frail older people such as affection and doing things that make you feel valued (Coast et al., 2008; Schuurmans, 2004) but more research is required, also on well‐being of informal caregivers.

Our systematic review indicates that we possibly need to shift our focus from effectiveness in terms of clinical outcomes to the *process* of integrated care. Integration implies “bringing together or merging the elements or components that were formerly separate” (Kodner & Spreeuwenberg, 2002) and integrated care is one strategy designed to solve the fragmentation of care, lack of continuity and co‐ordination (Fabbricotti, 2007; Kodner, 2009). However, our review shows that the focus of research is mainly on health and healthcare utilisation outcomes rather than on the care process. The evidence thus far on care process outcomes is rather promising. Consequently, professionals, researchers and policy makers might need to shift their expectations of the influence of integrated care from health outcomes to achieving organisational aims such as maintaining continuity and integrating health, social and informal care. This requires further empirical work on valid measurement instruments for the care process (see also Bautista, Nurjono, Lim, Dessers, & Vrijhoef, 2016), as well as on outcomes for the informal caregivers and professionals.

Future research should provide recommendations on specific cost drivers of preventive, integrated care for frail older people. Researchers considered various types of costs to determine the cost‐effectiveness of preventive, integrated interventions. There seems to be some consensus on the consideration of hospital care, nursing home admissions, home care and primary care but until now other types of care such as paramedical care and different forms of social care (psychosocial care, meals on wheels, day care) have often been neglected.

A final implication is that researchers might want to adopt a less static approach to research since both integration and frailty are dynamic, complex processes. The evaluations are summative; researchers have taken two to four quantitative snapshots in time. However, it might be useful to monitor both the frail older people and the integration process more closely and continuously. Integration is very complex since it involves overcoming several barriers to integration (Kodner, 2009; Valentijn et al., 2013). Close continuous monitoring would also lead to more transparency on the specific contents of the interventions, particularly the follow‐up, since the description of the interventions in the current type of evaluation research is limited (see also Eklund & Wilhelmson, 2009). Action research, which integrates research and practice in close co‐operation could be a future direction of study in order to improve daily care practice (Meyer, 2000).

## 5 CONCLUSION

The diverse and high expectations for preventive, integrated care for community‐dwelling frail older people in research, policy and practice should be tempered slightly. Our systematic review does not provide a solid base of evidence, particularly for important policy aims such as preventing functional decline and institutionalisation. Effectiveness may be pursued in other outcomes, such as well‐being and care processes. The level of integration is not decisive since higher level of integration does not seem to lead to better outcomes. More attention should be devoted to exploring effectiveness for subgroups of frail older people. Researchers in integrated care should be more aware of the underlying principles of the topic of integrated care: they should integrate their research, consider continuity and differentiate between frail older people.

## ACKNOWLEDGEMENTS

The authors thank Wichor Bramer, librarian from the Erasmus Medical Centre Rotterdam, for the help with the search terms and designing the search syntaxes for all nine databases.

## AUTHOR CONTRIBUTIONS

WL screened the abstracts, reviewed the full texts, assessed the risk of bias, extracted the data and wrote the paper. IF screened the abstracts and reviewed full texts that met the inclusion criteria or where doubts arose. RH assessed the risk of bias of the included studies. IF and RH have critically reviewed the content of the paper and contributed to revising the paper. All authors have approved the submitted version of the manuscript.
