# Supplementary material for: The (cost‐)effectiveness of preventive, integrated care for community‐dwelling frail older people: A systematic review
Source: Health Soc Care Community. 2018 Apr 17;27(1):1–30. doi: 10.1111/hsc.12571 (PMC7379491; doi:10.1111/hsc.12571)
Supplement: Supplementary file 1 [file HSC-27-1-s001.docx]

Supporting material table 1: Risk of bias

| Authors | 1. Was the allocation sequence adequately generated? | 2. Was the allocation adequately concealed? | 3. Were baseline outcome measure-ments similar? | 4. Were baseline characteristics similar? | 5. Was incomplete outcome data adequately addressed? | 6. Was knowledge of the allocated intervention adequately prevented during the study? | 7. Was the study adequately protected against contamination? | 8. Was the study free from selective outcomes reporting? | 9. Was the study free from other risks of bias? | Total EPOC- score |
| --- | --- | --- | --- | --- | --- | --- | --- | --- | --- | --- |
| Béland et al. 2006 | 1 | 1 | ? | 1 | 0 | 1 | 1 | 1 | 0 | 6 |
| Bleijenberg et al. 2014 | 1 | 1 | 1 | ? | ? | 0 | 1 | 1 | 0 | 5 |
| Drubbel et al. 2014 | ? | ? | ? | ? | 1 | ? | 1 | 1 | 0 | 3 |
| Burns et al. 1995 | 1 | 1 | 1 | 1 | 1 | 0 | 1 | 1 | 1 | 8 |
| Burns et al. 2000 | ? | ? | 1 | 1 | ? | 0 | 1 | 1 | 1 | 5 |
| Dalby et al. 2000 | 1 | 1 | ? | 1 | ? | 1 | 1 | 1 | 1 | 7 |
| de Stampa et al. 2014 | 0 | 0 | 1 | 1 | ? | ? | 0 | 1 | 1 | 4 |
| Ekdahl et al. 2016 | 1 | 1 | ? | 1 | ? | 1 | 0 | 1 | 0 | 5 |
| Engelhardt et al. 1996 | ? | ? | 1 | 1 | 0 | 1 | 1 | 1 | 1 | 6 |
| Toseland et al. 1996 | ? | ? | 1 | 1 | ? | 1 | ? | 0 | 1 | 4 |
| O'Donnell, Toseland 1997 | ? | ? | ? | 1 | ? | 1 | 1 | 1 | 1 | 5 |
| Fairhall et al. 2015 | ? | ? | 1 | 1 | 1 | 1 | 0 | 1 | 1 | 6 |
| Gagnon et al. 1999 | 1 | 1 | ? | 1 | 1 | 1 | 0 | 1 | 1 | 7 |
| Gray et al. 2010 | ? | ? | 1 | 1 | ? | ? | 0 | 1 | 0 | 3 |
| Hébert et al. 2008 | 0 | 0 | 0 | 0 | ? | 0 | 1 | 1 | 0 | 2 |
| Hébert et al. 2010 | 0 | 0 | 0 | 0 | ? | ? | 1 | 1 | 0 | 2 |
| Hinkka et al. 2007 | 1 | 1 | 1 | 1 | 1 | 1 | 0 | 0 | 1 | 7 |
| Kehusmaa et al. 2010 | 1 | 1 | ? | 1 | 1 | 1 | 0 | 1 | 1 | 7 |
| Kerse et al. 2014 | 1 | 1 | 1 | 1 | ? | 1 | 0 | 0 | 1 | 6 |
| Kono et al. 2012 | 1 | 1 | 0 | ? | 1 | ? | 0 | 1 | 1 | 5 |
| Kono et al. 2013 | 1 | 1 | ? | ? | ? | ? | 0 | 1 | 0 | 3 |
| Kono et al. 2016 | 1 | 1 | 1 | ? | ? | 1 | 0 | 1 | 1 | 6 |
| Kristenson et al. 2010 | 1 | 1 | 1 | 1 | ? | ? | 0 | 1 | 0 | 5 |
| Möller et al. 2013 | 1 | 1 | 1 | 1 | 1 | 0 | 0 | 1 | 1 | 7 |
| Sandberg et al. 2015a | 1 | 1 | 1 | 1 | 1 | 1 | 0 | 1 | 1 | 8 |
| Sandberg et al. 2015b | ? | ? | 1 | 1 | 1 | 1 | 0 | 1 | 1 | 6 |
| Leung et al. 2010 | 0 | 0 | ? | 1 | 1 | ? | 1 | 1 | 1 | 5 |
| Looman et al. 2014 | 0 | 0 | 1 | 0 | ? | ? | 1 | 1 | 1 | 4 |
| Makai et al. 2015 | 0 | 0 | ? | 0 | 1 | ? | 1 | 1 | 0 | 3 |
| Looman et al. 2016a | 0 | 0 | 1 | 0 | ? | ? | 1 | 1 | 1 | 4 |
| Looman et al. 2016b | 0 | 0 | ? | 0 | 1 | ? | 1 | 1 | 1 | 4 |
| Melis et al. 2008a | 1 | 1 | 1 | 1 | 1 | 0 | 1 | 1 | 1 | 8 |
| Melis et al. 2008b | ? | ? | 1 | 1 | 1 | ? | 1 | 1 | 1 | 6 |
| Metzelthin et al. 2013 | 1 | 1 | 1 | 0 | 1 | 1 | 1 | 1 | 1 | 8 |
| Metzelthin et al. 2015 | 1 | ? | 1 | 0 | ? | ? | 1 | 1 | 1 | 5 |
| Montgomery, Fallis 2003 | 1 | 1 | 0 | 0 | 0 | 1 | 0 | 0 | 0 | 3 |
| Morishita et al. 1998 | ? | ? | ? | 1 | ? | ? | 0 | 1 | 1 | 3 |
| Boult et al. 2001 | 1 | 1 | 1 | 1 | 1 | 1 | 1 | 1 | 1 | 9 |
| Reuben et al. 1999 | 1 | 1 | 1 | 1 | 1 | 1 | 1 | 1 | 1 | 9 |
| Rockwood et al. 2000 | ? | ? | 1 | 1 | ? | 1 | 1 | 1 | 0 | 5 |
| Rubenstein et al. 2007 | 1 | 1 | 1 | 1 | 1 | 1 | 1 | 0 | 1 | 8 |
| Ruikes et al. 2015 | 0 | 0 | 1 | 0 | 0 | 0 | 1 | 1 | 0 | 3 |
| Schreader et al. 2008 | 0 | 0 | 1 | 0 | 0 | ? | 1 | 1 | 0 | 3 |
| Shapiro Taylor 2002 | 1 | 1 | 1 | 1 | 0 | 0 | 0 | 1 | 1 | 6 |
| Tourigny et al. 2004 | 0 | 0 | 1 | 1 | ? | ? | 1 | 1 | 1 | 5 |
| van Leeuwen et al. 2015 | ? | ? | ? | 0 | 1 | ? | 1 | 1 | 0 | 3 |
