# Supplementary material for: The (cost‐)effectiveness of preventive, integrated care for community‐dwelling frail older people: A systematic review
Source: Health Soc Care Community. 2018 Apr 17;27(1):1–30. doi: 10.1111/hsc.12571 (PMC7379491; doi:10.1111/hsc.12571)
Supplement: Supplementary file 2 [file HSC-27-1-s002.docx]

Supporting Material Table 2: Intervention characteristics – components and levels of integration

Supporting material table 2a Short description and focus of intervention

| **Authors** | **Name intervention** | **Short description intervention** | **Focus intervention/ primary outcome** |
| --- | --- | --- | --- |
| Béland et al. 2006 | System of Integrated Care for Older Persons (SIPA) | Community-based care with local agencies responsible for the full range and coordination of community and institutional (acute and long-term) health and social services. | Meet the needs of the frail elderly, to assure comprehensive care, integration by all professionals and institutions involved |
| Bleijenberg et al. 2014, Drubbel et al. 2014 | U-PRIM & U-CARE | U-PRIM, a frailty screening intervention based on routine care data, and of U-PRIM followed by U-CARE, a nurse-led personalised care intervention. | Preserving daily functioning |
| Burns et al. 1995, Burns et al. 2000 | Interdisciplinary outpatient primary care Geriatric Evaluation and Management | After an initial comprehensive assessment, older veterans received long-term management in the geriatric clinic. | Focus on evaluation and long-term management, or primary care. |
| Dalby et al. 2000 | Preventive home visits by a nurse | A screening questionnaire identified eligible participants (those aged 70 years or more at risk of sudden deterioration in health). During preventive home visits by a nurse, patients were assessed and followed in their home for 14 months. | Minimize negative effects of age-related changes and risk factors and promote positive functional consequences |
| de Stampa et al. 2014 | Coordination Personnes Agées | Integrated primary care with intensive case management for community-dwelling, very frail elderly patients. | Better fit services and needs elderly, reduce excess healthcare use, improve continuity of care |
| Ekdahl et al. 2016 | Ambulatory Geriatric Assessment – a Frailty Intervention Trial (AGe-FIT) | Comprehensive Geriatrics Assessment-based care intervention characterized by home visits, participants visits to the ambulatory geriatric unit, and/or telephone calls, according to each participant's needs and preferences. | Prevent functional decline and managing main symptoms and diseases to improve health and quality of life. |
| Engelhardt et al. 1996, Toseland et al. 1996, O'Donnell, Toseland 1997 | Outpatient Geriatric Evaluation and Management in VAMC | Included an initial comprehensive assessment, the development and implementation of a care plan, periodic reassessment, monitoring and updating the care plan and referral to and coordination with other health and social service providers within and outside the VAMC. | Improve care and reduce utilization |
| Fairhall et al. 2015 | FIT intervention | A 12-month multifactorial, interdisciplinary intervention was individualized to each participant based on the frailty criteria present and incorporated the principles of geriatric evaluation and management. | Targeting identified frailty characteristics |
| Gagnon et al. 1999 | Nurse case management | Coordination and provision of healthcare services by nurses, both in and out the hospital, for a 10-month period. | Integrate care from a health maintenance and promotion perspective |
| Gray et al. 2010 | Anticipatory and Preventive Team Care (APTcare) | The central thrust of the intervention was to ensure evidence-based disease management and strong social supports to patients. | Ensure evidence-based disease management and strong social supports to patients |
| Hébert et al. 2008, Hébert et al. 2010 | Program of Research to Integrate the Services for the Maintenance of Autonomy (PRISMA) | Innovative coordination-type integrated service delivery system to improve continuity and increase the effectiveness and efficiency of services. PRIMA composes six components: coordination between decision makers and managers at the regional and local level, singe entry point, single assessment instrument coupled with case-mix management system, case management, individualized service plan and computerized clinical chart. | Increase the effectiveness and efficiency of services |
| Hinkka et al. 2007; Kehusmaa et al. 2010 | Network-based geriatric rehabilitation intervention | 3 in-patient periods at rehabilitation centres and a home visit by a professional. | Enabling living in community independently |
| Kerse et al. 2014 | Brief Risk Identification Geriatric Health Tool (BRIGHT) | The BRIGHT screening tool was sent to older adults every birthday; those with a score of 3 or higher were referred to regional geriatric services for assessment and, if needed, service provision. | Disability |
| Kono et al. 2012, Kono et al. 2013, Kono et al. 2016 | Preventive home visit model | Nurses and care managers provided structured preventive home visits every 6 months over 2 years, with a systematic assessment of care to prevent functional decline. | Locomotion, daily activities, social contacts or relationships with other people, health conditions, sign of abuse |
| Kristenson et al. 2010, Möller et al. 2013, Sandberg et al. 2015, Sandberg et al. 2015 | Case management programme | Case management programme with the focus on functional status and an important role of physiotherapist. Intervention was carried out by nurses and physiotherapists working as case managers who undertook home visits at least once a months and employed a multifactorial preventive approach. | Functional status |
| Leung et al. 2010 | Community-based Care Management Service (CMS) | Intervention provided by a care management team that comprised both professionals and paraprofessionals. | Fall prevention |
| Looman et al. 2014, Makai et al. 2015, Looman et al. 2016a, Looman et al. 2016b | Walcheren Integrated Care model (WICM) | Pro-active screening for frailty, assessment of care needs, GP practice as single entry point from which case management was provided, GP as coordinator of care, and the process was supported by multidisciplinary meetings, protocols and web-based files. | Improve quality of life |
| Melis et al. 2008a, Melis et al. 2008b | Dutch EASYcare Study Geriatric Intervention Programme (DGIP) | Starting off from a wide multidimensional assessment, the intervention team developed an individualized, integrated treatment plan for each patient. | Five problems: cognition, nutrition, behaviour, mood, mobility. |
| Metzelthin et al. 2013, Metzelthin et al. 2015 | Prevention of Care Approach | Multidimensional assessment and interdisciplinary care based on a tailor made treatment plan and regular evaluation and follow-up. | Participation in social and productive activities |
| Montgomery, Fallis 2003 | South Winnipeg Integrated Geriatric (SWING) | Multidimensional assessment, enhanced access to geriatric medical and day-hospital services and case management for 3 months period. | Impact on health care service utilization |
| Morishita et al. 1998, Boult et al. 2001 | Outpatient Geriatric Evaluation and Management | Comprehensive assessment followed by interdisciplinary primary care in a clinic from a team consisting of a geriatrician, a nurse, social worker and a gerontological nurse practitioner. | Functional ability, use of health service, satisfaction |
| Reuben et al. 1999 | Outpatient Comprehensive Geriatric Assessment consultation | A single outpatient Comprehensive Geriatric Assessment consultation coupled with an intervention to improve primary care physician and patient adherence with Comprehensive Geriatric Assessment recommendations. | Improve primary care physician and patient adherence with Comprehensive Geriatric Assessment recommendations. |
| Rockwood et al. 2000 | Mobile Geriatric Assessment Team | Three-month implementation of Comprehensive Geriatric Assessment recommendations by a Mobile Geriatric Assessment Team. | Goal attainment |
| Rubenstein et al. 2007 | Screening, Case Finding and Referral System for Older Veterans in Primary Care | The intervention combined a structured telephone geriatric assessment by a physician assistant, individualized referrals and recommendations, selected referral to outpatient geriatric assessment, and ongoing telephone case management. | Five geriatric target conditions: depression, cognitive impairment, urinary continence, falls, functional impairment |
| Ruikes et al. 2015 | CareWell | General practitioner-led extensive, multicomponent program integrating cure, care and welfare for the prevention of functional decline. | Prevention functional decline |
| Schreader et al. 2008 | Collaborative primary care nurse management intervention | Emphasising collaboration between physicians, nurses and patients, risk identification, comprehensive assessment, collaborative planning, health monitoring, patient education and transitional care. | Chronic illnesses/health care utilization and costs |
| Shapiro Taylor 2002 | Community-Based Early Intervention Program | An early interventive social service program designed to provide case-managed services earlier than clients would normally receive them to allow older adults to remain independent. | High quality of life and a lower risk of institutionalization and mortality |
| Tourigny et al. 2004 | Bois-Francs ISD network | Coordinated model where every organization keeps its own structure while adapting its operation to the agreed requirements and processes, and integrates all the gerento-geriatric services in the area, from promotion and prevention to diagnosis, treatment, rehabilitation, long-term care and palliative care. | Better planning and coordination for meeting individual needs |
| van Leeuwen et al. 2015 | Geriatric Care Model | Consisted of the following components: a regularly scheduled in-home comprehensive geriatric assessment by a practice nurse, followed by a customized care plan, management and training of practice nurse by a geriatric expert team, and coordination of care through community care network meetings and multidisciplinary team consultations of individuals with complex care needs. | Target health risks and care needs at an early stage, stimulate active involvement of older adults in the care process, improve coordination between professionals |

Supporting material table 2b Service integration of interventions

|  | **Service integration (micro-level)** |  |  |  |
| --- | --- | --- | --- | --- |
| **Authors** | **Assessment** | **Care plan** | **Follow-up** | **Single entry point** |
| Béland et al. 2006 | Yes, comprehensive |  | Case management by nurse or social worker: follow and intervene with patients & caregivers, liaise with family physicians, assure continuity, ease transitions, 24 hour on-call services | Yes |
| Bleijenberg et al. 2014, Drubbel et al. 2014 | Yes, two-stage: Frailty & bio-psychosocial needs; comprehensive (falls & mobility, physical functioning, nutrition and malnutrition, cognitive decline, polypharmacy, mood & depression, loneliness, vision problems & hearing loss, urinary incontinence, caregiver burden) | Yes, developed by practice nurse and GP | Interventions from evidence-based care plan for all ten health problems (e.g. polypharmacy: multifactorial interventions; tailored patient education, instruction, support, feedback and follow-up; tools and reminders for adherence) |  |
| Burns et al. 1995, Burns et al. 2000 | Yes, functional limitations, gait impairment, incontinence, polypharmacy, depression, and cognitive impairment, resources | Yes, developed by team | Follow-up in GEM clinic by most appropriate team member, who functions as liaison between participant and team |  |
| Dalby et al. 2000 | Yes, review of chart & additional comprehensive assessment (physical, cognitive, emotional & social function, medication use, the safety & suitability of home environment) | Yes, developed with primary care physician, the patient, the family, caregivers and other health professionals | Case management by nurse: integrate services and agencies into care plan, follow-up, monitor, promote health, provide psychosocial support |  |
| de Stampa et al. 2014 | Yes, comprehensive: health and social needs, preventive strategies | Yes, developed by case manager, approved with multidisciplinary team | Case management by nurse: implement care plan, care coordination, follow-up, re-assess needs every 3 months, contact with professionals, patient and family | Yes |
| Ekdahl et al. 2016 | Yes, comprehensive: medical, functioning, psychological, cognitive, social; for each discipline separately |  | Follow-up by home visits, telephone calls or visits to clinic according to participant's needs and preferences, at least 2 visits, obtain overall picture of life situation, team decides on further action (treatment within unit or referrals), available during office hours |  |
| Engelhardt et al. 1996, Toseland et al. 1996, O'Donnell, Toseland 1997 | Yes, comprehensive | Yes | Follow-up by GEM team: periodic assessment, monitoring and updating care plan, referral to and coordination with other health and social service providers, educate patient and informal caregiver, help with psychosocial and financial problems |  |
| Fairhall et al. 2015 | Yes, frailty characteristics (weight loss; exhaustion and Geriatric Depression Scale; social isolation; grip weakness, four metre walk time or physical activity level; self-efficacy, motivational readiness for change and goal appraisement; general health status, caregiver distress) |  | Specific follow-up and interventions for each frailty condition separately (e.g. referrals, home exercise program, medication review, supportive intervention care giver) |  |
| Gagnon et al. 1999 | Yes, current health status (physical, functional, social, & environmental aspects), review of the perceived needs of older person and caregiver | Yes | Case management by nurse: monitoring, follow-up every month, integrate care, support older people & caregiver, coordination work of all professionals, available at beepers |  |
| Gray et al. 2010 | Yes, review of record and additional assessment | Yes, developed by nurse practitioner and pharmacist, reviewed with family physician | Follow-up by nurse practitioner and pharmacist, education sessions with health-related information, home telehealth monitoring system for selection participants |  |
| Hébert et al. 2008, Hébert et al. 2010 | Yes, functional ability in ADL, mobility, communication, mental functions & IADL and resources | Yes, developed by case manager, approved in multidisciplinary meeting | Case management by nurse, social worker or other professional: planning of and admission to services, coordinate support & multidisciplinary team, advocate, monitor, reassess patient every 6 months | Yes |
| Hinkka et al. 2007; Kehusmaa et al. 2010 | Yes, comprehensive | Yes | Multidisciplinary group intervention on physical psychological and social activation, motivate to adopt an active lifestyle, classes on disease management and coping strategies, and recreational activities |  |
| Kerse et al. 2014 | Yes, comprehensive |  | Coordination of support rehabilitation services & geriatrical medical expertise |  |
| Kono et al. 2012, Kono et al. 2013, Kono et al. 2016 | Yes, assessment of locomotion, daily activities, social contacts or relationships with other people, health conditions, signs of abuse | Yes | Home visits by community care nurses, care managers or social workers every 6 months, evaluate care plan |  |
| Kristenson et al. 2010, Möller et al. 2013, Sandberg et al. 2015, Sandberg et al. 2015 | Yes, comprehensive: Functional status, physical function, vibration sensations. | Yes | Case management by nurse or physiotherapist: care coordination, follow-up visits every month, advocacy, providing general and specific information, safety, available during working hours |  |
| Leung et al. 2010 | Yes, comprehensive | Yes | Providing falls prevention interventions, providing/linking elders and caregivers for services, review care plan & progress by professionals such as social workers, nurses, and physiotherapists |  |
| Looman et al. 2014, Makai et al. 2015, Looman et al. 2016a, Looman et al. 2016b | Yes, comprehensive: Activities daily life, cognition, mood, support care givers | Yes, formulated consultation with frail older people and informal caregiver, developed by case manager, approved in multidisciplinary meeting | Case management by practice nurse: coordinate care, monitoring, admittance to services, contact person for professionals, evaluating treatment plan, follow-up at least every 6 months | Yes |
| Melis et al. 2008a, Melis et al. 2008b | Yes, multidimensional |  | Follow-up visits by geriatric specialist nurse for additionals geriatric evaluation and management |  |
| Metzelthin et al. 2013, Metzelthin et al. 2015 | Yes, multidimensional: Problems daily activities, risk factors daily activities | Yes, formulated with frail older person | Case management by practice nurse: executing treatment plan with intervention protocol and toolbox of interventions, evaluation of achievement of goals, implementation of strategies in daily life, need of support in the following period, update other professionals |  |
| Montgomery, Fallis 2003 | Yes, comprehensive: History, functional, social and environmental | Yes, formulated by coordinator, reviewed by geriatrician & day-hospital team | Case management for 3 months: provide resources, resolve problems, preventive measures, referrals to home and community-based services |  |
| Morishita et al. 1998, Boult et al. 2001 | Yes, comprehensive: medical conditions, psychosocial status, functional ability, cognitive status, nutritional risk, use of alcohol, social network, gait and balance, environmental safety, medications, advance directives, hearing and vision | Yes, developed by EM nurse, social worker, and geriatrician | Case management by team of geriatrician, a nurse, social worker and a gerontological nurse practitioner: diagnose and treat problems, adjust medication regime, provide counselling and health education, referrals to other services, assistance with directives, monthly visits to clinic, 24-hour on-call services |  |
| Reuben et al. 1999 | Yes, comprehensive, for each discipline separately |  | Adherence intervention of assessment for patients and their physicians, ensure understanding of recommendations, assess level of agreement, empower patient to interact proactively with physician to implement and adhere recommendations |  |
| Rockwood et al. 2000 | Yes, comprehensive: mental status, emotional health, communication, mobility, balance, bowels, bladders, nutrition, daily activities, social situation | Yes, developed by geriatric nurse assessor and geriatrician |  |  |
| Rubenstein et al. 2007 | Yes, two stage: Assessment of specific risk and unmet needs; evaluation of physical health, functional status, mental health, and social and environmental status | Yes | Case management by physician assistant with supervision geriatrician: coordinate follow-up, follow-up every three months, refer to services, health promotion recommendations & health education, monitor |  |
| Ruikes et al. 2015 | Yes | Yes, revised during team meetings | Case management by nurse or social worker: coordinate and monitor care, plan team meetings, acknowledge participants of care plan, involve participants in settings goals, maintain contact with participants and informal caregiver, hold a medication review (by ≥5 chronically prescribed drugs) |  |
| Schreader et al. 2008 | Yes, comprehensive | Yes, developed by nurse case manager and primary care physician | Case management by nurse and care assistant: review and update care plan, monitor health status, identify adherence to treatment regime, provide ongoing health education on managing health, coordination and arrange health-related services, follow-up every month |  |
| Shapiro Taylor 2002 | Yes, geriatric |  | Case management: care planning, monitoring, prescribe and coordinate services, follow-up every 3 months |  |
| Tourigny et al. 2004 | Yes, physical and psychological health, social aspects, and functional autonomy. | Yes | Case management by professionals trained in social services for most complex cases | Yes |
| van Leeuwen et al. 2015 | Yes comprehensive | Yes, developed by nurse with primary care physician | Guideline-concordant management and treatment options, involve older adults in decision-making process, evaluate of care plan |  |

Supporting material table 2c Professional, organizational and system integration of interventions

|  | **Professional integration (meso-level)** | | |  | **Organizational integration (meso-level)** | **System integration (macro-level)** |
| --- | --- | --- | --- | --- | --- | --- |
| **Authors** | **Focal organisation** | **Role GP** | **Team composition** | **Education professionals** | **Organizational integration** | **Financial integration** |
| Béland et al. 2006 | Network of organizations with one single entry point | Continues usual care, liaises with case manager, develops and applies protocols, working agreement between primary care professionals & interdisciplinary team | Multidisciplinary team: case managers, community nurses, social workers, occupational therapists, psychotherapists, homemakers, staff family physician, consultant pharmacists, community organizers |  | New organization - consortium of public institutions, agreements about service provision with other providers, e.g. hospital and long-term care institutions | Yes - Integration of all public financing for health and social services, teams controlled own budget |
| Bleijenberg et al. 2014, Drubbel et al. 2014 | GP practice |  | GP, practice nurse | Practice nurses: intervention training program 5 weeks of 4 hours on frailty assessment, content assessment, evidence-based care plans. GP and practice nurse: intervention training session 4 hours on content intervention |  |  |
| Burns et al. 1995, Burns et al. 2000 | GEM outpatient clinic |  | Interdisciplinary team: including physicians, nurse practitioner, social worker, psychologists, clinical pharmacists | All professionals: extensive training and development |  |  |
| Dalby et al. 2000 |  |  |  |  |  |  |
| de Stampa et al. 2014 | Primary care practice | Part of core team: care management, responsible for medical decision making | Two persons team - case manager and primary care physician, collaboration with geriatrician |  | Non-profit consortium: managers from community-based services and hospital setting |  |
| Ekdahl et al. 2016 | Ambulatory geriatric unit |  | Interdisciplinary team: doctors, nurses, psychotherapists, occupational therapist, dietician, social worker, pharmacist |  |  | No - Intervention was financed by Swedish government |
| Engelhardt et al. 1996, Toseland et al. 1996, O'Donnell, Toseland 1997 | Veterans Affair Medical Centre |  | GEM team: geriatrician, nurse practitioner, social worker |  |  |  |
| Fairhall et al. 2015 |  | Sub-optimal medication is discussed with GP | Interdisciplinary team: physiotherapists, geriatrician, rehabilitation physician, dietician, nurse. Referrals to psychiatrist, psychologist, day activity group, volunteer, and contact with GP about medication. |  |  |  |
| Gagnon et al. 1999 |  | Part of interdisciplinary team | Interdisciplinary team: case managers, community-based family physicians, psycho-geriatricians or psychologists, social workers, occupational therapists, psychotherapists, dieticians. Consultation from geriatricians, family physicians, staff physicians |  |  |  |
| Gray et al. 2010 | Community practice | Part of team | Physicians, nurses, support staff, nurse practitioners, pharmacists, family physicians |  |  |  |
| Hébert et al. 2008, Hébert et al. 2010 | Network of organizations with one single entry point | Main medical practitioner, primary collaborators of case manager, access to and coordination of specialised medical services | Multidisciplinary team | Case managers: special training | Network with Joint Governing Board of all health and social organisations and community agencies (public, private, and voluntary), coordination between decision makers and managers at the regional and local level | Limited - System funding as part of agreement between organizations |
| Hinkka et al. 2007; Kehusmaa et al. 2010 | Rehabilitation centre |  | Key members: rehabilitation team: physician, physiotherapist, social worker, occupational therapist |  | Network of organizations: rehabilitation institutes, local social and health providers, SII, non-governmental organizations |  |
| Kerse et al. 2014 | Primary care practice | Maintains responsibility for overall medical care | Multidisciplinary team: physiotherapists, occupational therapists, gerontology nurse, geriatrician, and social worker | Primary care practices: intervention processes, feedback about health and support services decision |  | Limited - Practices were funded for 1 day per month of a practice nurse's salary to complete BRIGHT recall process, and regional geriatrics services were bulk funded to provide assessment services to trial participants |
| Kono et al. 2012, Kono et al. 2013, Kono et al. 2016 | Community-based comprehensive care centres |  | Community health nurses, care managers, social workers |  |  |  |
| Kristenson et al. 2010, Möller et al. 2013, Sandberg et al. 2015, Sandberg et al. 2015 | Physio-therapists | Supports case manager | Nurse, physiotherapist, primary care physicians, hospital-based geriatric specialist |  |  |  |
| Leung et al. 2010 |  |  | Social workers, nurses, physiotherapists |  |  |  |
| Looman et al. 2014, Makai et al. 2015, Looman et al. 2016a, Looman et al. 2016b | Network of organizations with one single entry point | Coordinator of care, partner in prevention, single entry point | GP, nurse practitioner, second-line geriatric nurse practitioner, geriatric physiotherapists, geriatricians, pharmacists, district nurse, nursing home doctors, mental health workers | GP: training in geriatric care, assessment tool, GP consults. Case manager: training assessment tool, course case management | Network with Joint Governing Board of all involved organizations (GP practices, home care organizations, and nursing homes) |  |
| Melis et al. 2008a, Melis et al. 2008b |  | Continues usual care - primarly responible, referrals, medication changes and other interventions | Geriatric specialist nurse, primary care physician, geriatrician. Consultation of other involved health care workers, eg home care or physical therapist |  |  |  |
| Metzelthin et al. 2013, Metzelthin et al. 2015 | GP practice | Member core team | Core team: General practitioner and practice nurse. Close cooperation: occupational therapists and physical therapists, other professionals (pharmacists, geriatrician) | All professionals: training sessions and meetings on intervention protocol (e.g. screening, assessment), possibility to gain experience with protocol in practice |  |  |
| Montgomery, Fallis 2003 | Home care organization |  | Coordinator, geriatrician, day-hospital team |  |  |  |
| Morishita et al. 1998, Boult et al. 2001 | GEM-clinic | Continues usual care - GEM clinic coexists alongside regular primary care | Core team: geriatrician, nurse, social worker, gerontological nurse practitioner | All clinical staff: weekly seminars on topics as dementia, urinary incontinence |  |  |
| Reuben et al. 1999 |  | Adherence intervention for primary care physician | Board-certified geriatricians, nurse practitioner, social worker, physical therapist, health educator | Physicians: focus groups, one at a fee-for-service hospital and one at the a health maintenance organization hospital to develop the intervention |  |  |
| Rockwood et al. 2000 |  |  | Geriatric nurse assessors, geriatricians, physiotherapists, occupational therapists, social worker, dietitian, audiologist, speech-language pathologist |  |  |  |
| Rubenstein et al. 2007 | Geriatric assessment clinic | Continues care as usual -results assessment and recommendations are reported to primary care provider | Case manager, geriatrician, geriatric medicine faculty, physician assistant, internal medicine house staff, geriatric psychiatrist, geriatrician with expertise in incontinence, physical therapist | Geriatric assessment clinic is an assessment and teaching clinic |  |  |
| Ruikes et al. 2015 | GP practice |  | Core multidisciplinary team: GP, practice nurse or community nurse, elderly care physician, social worker | Persuasive communication and social influencing for participation, provision of additional information through a website, newsletters and written instructions, providing feedback and advice to the participating professionals |  | No - Financial reimbursement for all health care professionals and organizations to cover the extra efforts required by the program, to facilitate participation in the intervention |
| Schreader et al. 2008 | Primary care practice | Addition of registered nurse to the primary care practice |  | Primary care practices, nurses, administrators: measuring and reporting major study outcomes |  |  |
| Shapiro Taylor 2002 |  |  | Geriatric nurse |  |  |  |
| Tourigny et al. 2004 | Network of organizations with one single entry point |  | No multidisciplinary team but integration of all the geronto-geriatric services in the area, from promotion and prevention to diagnosis, treatment, rehabilitation, long-term care, palliative care |  | Network with Joint Governing Board of managers of public institutions and community agencies | Limited - Pooling resources from four institutions for case management |
| van Leeuwen et al. 2015 | Primary care practice | Part of team | Practice nurse, GP, geriatric expert team (experienced geriatric nurse and an elderly care physician), pharmacists, other health care professionals could be consulted | Practice nurse: motivational interviewing course, workshop on assessment tool, management and training by expert team. Geriatrician: workshop on assessment tool | Regional network of organizations: providers of care services for older adults, primary care professionals, community-based organizations |  |

Supporting material table 2d Functional and normative integration of interventions and role informal caregiver and prevention in interventions

|  | **Functional integration** |  | **Normative integration** |  |  |
| --- | --- | --- | --- | --- | --- |
| **Authors** | **Coordination** | **Information system** | **Normative integration** | **Role informal caregiver** | **Prevention** |
| Béland et al. 2006 | Series of evidence-based interdisciplinary protocols (nutrition, falls, congestive heart failure, dementia, depression, medication, vaccination) |  |  | Encourage family participation in care and decision making, follow-up and intervene with patients & caregivers | Assessment; follow-up; SIPA is responsible for prevention (among other services) |
| Bleijenberg et al. 2014, Drubbel et al. 2014 | Flowchart with suggested interventions |  | Workshop about collaboration between GP’s and practice nurses | Assessment of caregiver burden | Screening, assessment; care plan; follow-up; screening intervention on falls/mobility, exercise programs that consist of muscle strengthening, balance retraining, endurance and flexibility, motivation, feedback, patient education on physical functioning; screening on nutritional status, patient education on loneliness |
| Burns et al. 1995, Burns et al. 2000 |  |  | Members of this team had worked together on an outpatient GEM clinic for approximately 3 years |  | Assessment; care plan; follow-up |
| Dalby et al. 2000 |  |  |  |  | Screening; assessment; care plan; follow-up; health promotion by case manager |
| de Stampa et al. 2014 | Interdisciplinary evidence-based protocols |  |  | Case managers can be reached by family members, support available from psychologists | Assessment (including preventive strategies); care plan; follow-up |
| Ekdahl et al. 2016 | Team conferences twice a week | Medical record |  | Next of kin are welcome as part of care team, services directed to informal caregiver, holistic appraisal of best course of action toward an increased quality of life of the patient | Assessment; follow-up |
| Engelhardt et al. 1996, Toseland et al. 1996, O'Donnell, Toseland 1997 | Weekly meetings GEM team | Customized software program linked to hospital mainframe computer |  | Social workers help caregivers with psychosocial and financial problems. Nurse practitioner educated informal caregiver | Assessment; care plan; follow-up; educate patient and informal caregiver |
| Fairhall et al. 2015 | Weekly interdisciplinary case-conferences, separate guidelines/interventions for each frailty characteristic |  |  | Supportive intervention when informal caregiver experiences significant distress | Assessment; follow-up |
| Gagnon et al. 1999 | Weekly meetings case manager and investigative team members | Computerized patient record |  |  | Assessment; care plan; follow-up |
| Gray et al. 2010 | Scheduled case conferences | Charting and electronical messaging, home health monitor system |  |  | Assessment; care plan; follow-up; education sessions to provide specific health related information. education and self-care as one of the five priorities |
| Hébert et al. 2008, Hébert et al. 2010 | Multidisciplinary meetings | Computerized clinical charts allowing communication between institutions and clinicians for monitoring purposes |  | Care plan is validated with informal caregivers to empower them in decision-making process | Assessment; care plan; follow-up |
| Hinkka et al. 2007; Kehusmaa et al. 2010 |  |  |  |  | Assessment; care plan; physical, psychological and social activation and counselling, motivating the participants to adopt an active lifestyle, classes on disease management and coping strategies, and recreational activities |
| Kerse et al. 2014 |  |  |  |  | Screening; assessment |
| Kono et al. 2012, Kono et al. 2013, Kono et al. 2016 | Rigorous recommendations for each of the five care needs categories |  |  |  | Assessment; care plan; follow-up; focus on preventing functional decline |
| Kristenson et al. 2010, Möller et al. 2013, Sandberg et al. 2015, Sandberg et al. 2015 | Meetings between nurses, physiotherapists and research group |  |  |  | Assessment; care plan; follow-up; physiotherapists had preventive approach |
| Leung et al. 2010 | Interventions based on a clinical protocol by multidisciplinary action |  |  |  | Assessment; care plan, fall prevention interventions |
| Looman et al. 2014, Makai et al. 2015, Looman et al. 2016a, Looman et al. 2016b | Multidisciplinary meetings, multidisciplinary protocols, web-based files, task reassignment and delegation between nurses and doctors and among GPs, nursing home doctors and geriatricians. Consultation among primary, secondary and tertiary care providers occurred | Web-based files |  | Explicit attention for support and guidance and informal caregiver, validate care plan with informal caregiver | Screening; assessment; care plan; follow-up; GP is partner in prevention |
| Melis et al. 2008a, Melis et al. 2008b | Interdiscplinary consults, separate guidelines for five health problems |  |  | Care burden assessment, results implemented in care plan | Assesment; follow-up |
| Metzelthin et al. 2013, Metzelthin et al. 2015 | Bilateral or extended team meetings, toolbox of interventions: enhancing meaningful activities, daily physical activity, social network and social activities, adapting environment, activities or skills, and stimulating health |  | Training sessions and meetings on client-centeredness, interdisciplinary collaboration | Support of social and physical environment, involvement in decision making and cooperative working relationship | Screening; assessment; care plan; follow-up; stimulating health is one of five topics in toolbox of interventions |
| Montgomery, Fallis 2003 |  |  |  |  | Assessment; care plan; follow-up with preventive measures |
| Morishita et al. 1998, Boult et al. 2001 | Daily meetings GEM team, clinical guidelines, for health maintenance, dementia, depression, urinary continence, constipation, osteoarthritis, and diabetes |  | All clinical staff attended weekly seminars on health maintenance. All staff attended a 8 hour workshop on team development |  | Assessment; care plan; follow-up; counselling and health education by team |
| Reuben et al. 1999 | Short interdisciplinary case conferences after evaluation |  |  |  | Screening; assessment; health educator contacted patient; empower patient for interaction physician |
| Rockwood et al. 2000 |  |  |  |  | Assessment; care plan |
| Rubenstein et al. 2007 | Interdisciplinary team meeting |  |  |  | Screening; assessment; care plan; follow-up; health promotion recommendation and health education by case manager |
| Ruikes et al. 2015 | Team meetings every 4 - 8 weeks, multidisciplinary guidelines on 8 common geriatric syndromes, a guideline on advance care planning, procedure agreement for easy-to-access consultation by geriatric experts, and procedure agreement between primary and specialized providers upon hospitalization and discharge | Web-based health and welfare portal | Tailor-made meetings, coaching on the job, helpdesk, and expert meetings to overcome gaps in knowledge, attitude and skills needed to conduct the program |  | Assessment; care plan; follow-up |
| Schreader et al. 2008 | Formal meetings, monthly reports of individual patients and their characteristics |  | Education series for primary care professionals, nurses and administrators on goals and responsibilities of the collaborative care teams and clinical decision making with older adult patients |  | Screening; assessment; care plan; follow-up; ongoing education on specific health problems and conditions by case manager |
| Shapiro Taylor 2002 |  |  |  | Care givers participated in care planning. | Assessment; follow-up; early interventive social service program designed to provide case-managed services earlier than clients would normally receive them to allow older adults to remain independent |
| Tourigny et al. 2004 |  | Geronto-geriatric information system by computerized clinical |  | Case management for complex cases - also caregivers. | Assessment; care plan; follow-up; integration of all the geronto-geriatric services in the area, including promotion and prevention |
| van Leeuwen et al. 2015 | Guideline-concordant management and treatment options | Digital patient system, tasks geriatric expert team: (quality) management, expert knowledge transfer by team meetings, training sessions and multidisciplinary patient reviews |  |  | Assessment; care plan; follow-up |
