# Supplementary material for: The (cost‐)effectiveness of preventive, integrated care for community‐dwelling frail older people: A systematic review
Source: Health Soc Care Community. 2018 Apr 17;27(1):1–30. doi: 10.1111/hsc.12571 (PMC7379491; doi:10.1111/hsc.12571)
Supplement: Supplementary file 3 [file HSC-27-1-s003.docx]

**Appendix Search strategy Embase**

('integrated health care system'/exp OR 'managed care'/exp OR 'case manager'/exp OR 'case management'/exp OR 'disease management'/de OR (((integrat* OR managed OR continuit* OR shared OR coordinat* OR chain* OR partnership*) NEAR/6 (care OR healthcare* OR system)) OR ((case* OR care OR discharge* OR comprehensiv* OR disease*) NEAR/3 manage*) OR ((Patient OR person) NEXT/1 (Centered* OR Centred*)) OR ((vertical* OR clinical* OR functional*) NEAR/3 integrat*)):ab,ti) AND ('frail elderly'/exp OR 'very elderly'/exp OR ('vulnerable population'/exp AND aged/exp) OR (frail* OR ((vulnerable OR very OR 'high risk') NEAR/3 (elder* OR old*)) OR 'oldest old' OR septagenar* OR octagenar* OR nonagenar* OR centenar* OR supercentenar*):ab,ti) AND ((family OR physician* OR practice*:de,it,lnk,ab,ti OR ‘primary care’ OR ‘Primary Health Care’/exp OR primary:de,it,lnk,ab,ti OR (general NEXT/1 pract*) OR gp:ab,ti OR gps:ab,ti) OR 'primary health care'/exp OR 'general practitioner'/exp OR 'general practice'/exp OR 'family medicine'/exp OR 'ambulatory care'/exp OR 'outpatient care'/exp OR 'outpatient department'/exp OR 'community care'/exp OR 'home care'/exp OR population/exp OR home/exp OR 'rural population'/exp OR 'urban population'/exp OR 'visiting nursing service'/exp OR 'homebound patient'/exp OR 'independent living'/exp OR (((primary OR 'first line') NEAR/3 (care OR healthcare)) OR ((general OR family) NEAR/3 (practitioner* OR practice* OR doctor* OR medicine* OR physician*)) OR gp OR gps OR ambulatory* OR outpatient* OR communit* OR ((living OR care OR healthcare OR management OR visit*) NEAR/6 home) OR (independent* NEAR/3 (living OR live)) OR 'home nursing' OR neighbo* OR population* OR 'nursing service' OR (visit* NEAR/3 nurs*) OR homebound OR (house NEXT/1 call*) OR 'Aging in Place'):ab,ti) AND [english]/lim NOT ([Conference Abstract]/lim OR [Letter]/lim OR [Note]/lim OR [Editorial]/lim)
